# Macrophage WEE1 Directly Binds to and Phosphorylates NF‐κB p65 Subunit to Induce Inflammatory Response and Drive Atherosclerosis

**DOI:** 10.1002/advs.202503192

**Published:** 2025-04-09

**Authors:** Zhuqi Huang, Sirui Shen, Weixin Li, Mengyang Wang, Yudie Yang, Wu Luo, Xue Han, Zheng Xu, Julian Min, Xiaohong Long, Weijian Huang, Gaojun Wu, Yi Wang, Guang Liang

**Affiliations:** ^1^ Department of Pharmacy and Institute of Inflammation Zhejiang Provincial People's Hospital Affiliated People's Hospital Hangzhou Medical College Hangzhou Zhejiang 310014 China; ^2^ School of Pharmacy Hangzhou Normal University Hangzhou Zhejiang 311000 China; ^3^ Key Laboratory of Cardiovascular Intervention and Regenerative Medicine of Zhejiang Province Department of Cardiology Sir Run Run Shaw Hospital Zhejiang University School of Medicine Hangzhou Zhejiang 310016 China; ^4^ Department of Cardiology the First Affiliated Hospital Wenzhou Medical University Wenzhou Zhejiang 325035 China; ^5^ School of Pharmaceutical Sciences Hangzhou Medical College Hangzhou Zhejiang 311399 China

**Keywords:** atherosclerosis, inflammation, macrophage, NF‐κB p65, WEE1

## Abstract

Atherosclerosis has an urgent need for new therapeutic targets. Protein kinases orchestrate multiple cellular events in atherosclerosis and may provide new therapeutic targets for atherosclerosis. Here, a protein kinase, WEE1 G2 checkpoint kinase (WEE1), promoting inflammation in atherosclerosis is identified. Kinase enrichment analysis and experimental evidences reveal macrophage WEE1 phosphorylation at S642 in human and mouse atherosclerotic tissues. RNA‐seq analysis, combined with experiment studies using mutant WEE1 plasmids, shows that WEE1 phosphorylation, rather than WEE1 expression, mediated oxLDL‐induced inflammation in macrophages. Macrophage‐specific deletion of WEE1 or pharmacological inhibition of WEE1 kinase activity attenuates atherosclerosis by reducing inflammation in mice. Mechanistically, RNA‐seq and co‐immunoprecipitation followed by proteomics analysis are used to explore the mechanism and substrate of WEE1. p‐WEE1 promoted inflammatory response through activating NF‐κB shown and further revealed that WEE1 can directly bind to the p65 subunit. It is confirmed that p‐WEE1 directly interacts with the RHD domain of p65 and phosphorylates p65 at S536, thereby facilitating subsequent NF‐κB activation and inflammatory response in macrophages. The findings demonstrate that macrophage WEE1 drives NF‐κB activation and atherosclerosis by directly phosphorylating p65 at S536. This study identifies WEE1 as a new upstream kinase of p65 and a potential therapeutic target for atherosclerosis.

## Graphical abstract

The present study shows that macrophage WEE1 drives NF‐κB activation and inflammatory atherosclerosis by directly phosphorylating p65 at S536. This finding illustrates a macrophage‐specific WEE1‐p65 axis in regulating inflammatory atherosclerosis and points out new directions to broaden the clinical applications for WEE1 inhibitors in atherosclerosis and even other inflammatory diseases.

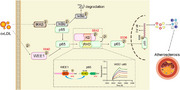

## 1 Introduction

Atherosclerosis is a major cardiovascular disease characterized by high morbidity and mortality rates worldwide.^[^ ^1^ ^]^ The chronic inflammatory response mediated by macrophages in the atherosclerotic lesions is one pivotal step in the evolution of atherosclerosis.^[^ ^2^ ^]^ When low‐density lipoproteins (LDL) accumulate and activate the vascular endothelium, macrophages are recruited to atherosclerotic lesions and release a significant amount of proinflammatory factors to promote atherosclerosis development.^[^ ^3^ ^]^ Infiltrated macrophages also form macrophage‐like foam cells, accumulating in the subendothelial space to develop a necrotic core. Consequently, the identification of novel regulatory molecules involved in macrophage inflammation and elucidation of their roles in atherosclerosis may provide potential targets for the treatment of this disease.

Protein kinases (PKs) modulate a wide range of cellular events through phosphorylating the substrate proteins and are considered important targets for basic research and drug development. In the past decades, PK inhibitors have proven efficacy for the treatment of many oncological disorders. PKs also orchestrate a wide range of cellular events in both physiological homeostasis and pathological states in cardiovascular diseases including atherosclerosis.^[^ ^4^ ^]^ Recent publications on the involvement of novel PKs in atherosclerosis have enhanced our knowledge. Mammalian sterile 20‐like kinase 1,^[^ ^5^ ^]^ liver kinase B1,^[^ ^6^ ^]^ and mechanistic target of rapamycin kinase,^[^ ^7^ ^]^ have been investigated to regulate the atherosclerosis pathology. Therefore, identifying key PKs involved in atherosclerosis may provide new therapeutic targets and drug development for atherosclerosis.

Since PKs function by the pro‐phosphorylating enzyme activity, PKs may predominantly undergo alterations in the kinase activity while maintaining unchanged total expression levels in the pathological state of diseases.^[^ ^8^ ^]^ Kinase enrichment analysis (KEA) of differentially expressed genes (DEGs) from the RNA‐sequencing data provides a good way to identify the activity‐changed PKs associated with diseases.^[^ ^9^ ^]^ In the present study, we profiled activity‐changed PKs in atherosclerotic aortas of mice compared with normal aortas using RNA‐Seq‐based KEA and identified an activity‐increased PK, WEE1 G2 checkpoint kinase (WEE1), in atherosclerotic lesions. WEE1 is a serine/threonine kinase recognized as a cell cycle regulator in normal cell cycle and DNA damage.^[^ ^10^ ^]^ WEE1 plays a crucial role in the G2‐M cell‐cycle checkpoint arrest for DNA repair before mitotic entry.^[^ ^10^ ^]^ Abundant studies have shown a range of WEE1 activities in human tumorigenesis, such as hepatocellular carcinoma,^[^ ^11^ ^]^ breast cancers,^[^ ^12^ ^]^ and squamous cell carcinoma.^[^ ^13^ ^]^ Therefore, WEE1 is considered as a potential anti‐cancer target and the small‐molecule WEE1 inhibitors have been evaluated for pre‐clinical cancer treatment.^[^ ^14^ ^]^ In addition, WEE1 has been recently reported to promote endometriosis via the Wnt/β‐catenin signaling pathway.^[^ ^15^ ^]^ However, the role of WEE1 in atherosclerosis and other cardiovascular diseases has not yet been studied.

The purpose of this study was to clarify the regulatory role and mechanism of WEE1 in atherosclerosis. We showed that macrophage WEE1 phosphorylation, but not protein level, positively correlated with high‐fat diet (HFD)‐induced atherosclerosis. Either macrophage WEE1 deficiency or inactivation significantly prevented atherosclerosis by reducing macrophage inflammation. Mechanistically, we demonstrated that WEE1 directly interacts with NF‐κB p65 subunit to phosphorylate p65 at S536, inducing subsequent NF‐κB activation and inflammatory gene transcription in macrophages. Our study illustrates a macrophage‐specific WEE1‐p65 axis in regulating atherosclerosis and indicates WEE1 as a pharmacological target for atherosclerosis.

## 2 Results

### 2.1 Macrophage p‐WEE1 is Up‐Regulated in Atherosclerotic Plaques

**Figure 1:**
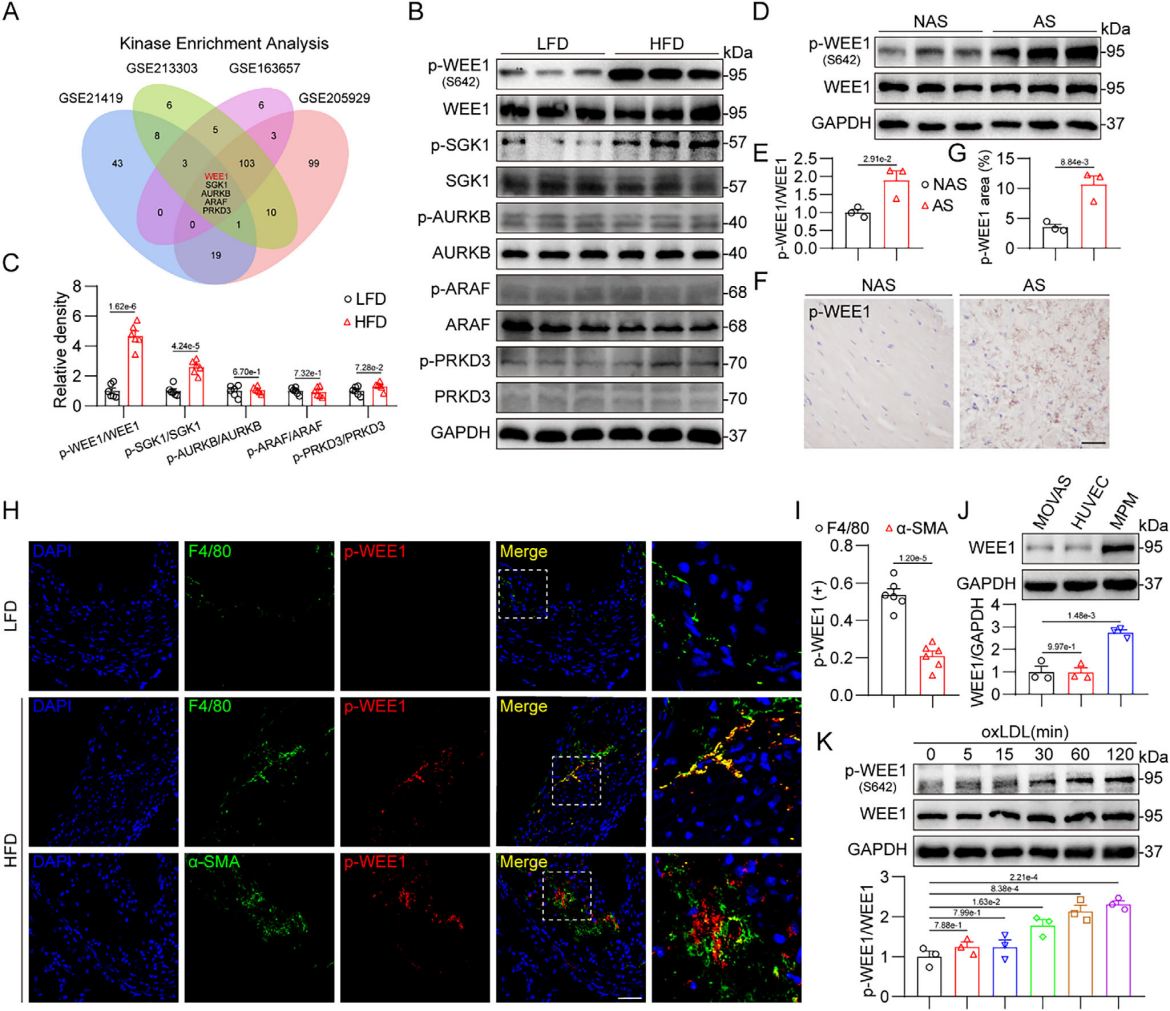
Macrophage p‐WEE1 is up‐regulated in atherosclerotic plaques. A) Venn diagram representing kinase enrichment analysis in atherosclerotic aortas compared to normal aortas from 4 public mouse datasets (GSE21419, GSE213303, GSE163657, and GSE205929). B,C) Western blot analysis B) and densitometric quantification C) of p‐WEE1, p‐SGK1, p‐AURKB, p‐ARAF, p‐PRKD3 in aortas of LFD and HFD‐fed ApoE^−/−^ mice. GAPDH, WEE1, SGK1, AURKB, ARAF and PRKD3 were used as loading controls (*n* = 6). D,E) Western blot analysis D) and densitometric quantification E) of p‐WEE1 in human non‐atherosclerotic carotid artery tissues (NAS) or atherosclerotic carotid artery tissues (AS) from three atherosclerotic patients. GAPDH and WEE1 were used as loading controls (*n* = 3). F,G) Representative immunohistochemistry staining F) and quantification G) of p‐WEE1 in NAS or AS from three atherosclerotic patients (*n* = 3, scale bar = 25 µm). H,I) Representative immunofluorescence staining H) and quantification I) of F4/80 (green) or α‐SMA (green) and p‐WEE1 (red) in aortic roots (*n* = 6, scale bar = 50 µm). J) Western blot analysis and densitometric quantification of WEE1 in mouse aortic vascular smooth muscle cell (MOVAS), human umbilical vein endothelial cell (HUVEC), and mouse primary peritoneal macrophage (MPM). GAPDH was used as the loading control (*n* = 3). K) Time‐course of p‐WEE1 induction in response to oxLDL in mouse primary peritoneal macrophages (MPMs). MPMs were exposed to oxLDL (50 µg mL^−1^) for indicated time. Western blot analysis and densitometric quantification of p‐WEE1 were shown. GAPDH was used as the loading control (*n* = 3). Data were shown as mean ± SEM. C, E, G, and I, Student's *t*‐test; J,K, one‐way ANOVA followed by Tukey's test.

To determine the potential protein kinases involved in atherosclerosis, we analyzed four publicly available transcriptome datasets from atherosclerotic mouse models using RNA‐seq data‐based kinase enrichment analysis (KEA). KEA identified five potential protein kinases (WEE1, SGK1, AURKB, ARAF, and PRKD3) whose kinase activities were predicted to be changed in atherosclerotic aortas compared to normal aortas in all four datasets (**Figure** **1**A). We built an atherosclerotic model using ApoE^−/−^ mice fed with HFD for 16 weeks, and then we examined the phosphorylation levels of these five kinases in the aorta tissues of mice. Among these five kinases, only WEE1 and SGK1's phosphorylations were remarkably increased in the aortas of HFD‐fed ApoE^−/−^ mice, while the total protein levels of WEE1 and SGK1 did not alter (Figure 1B,C). The RNA‐seq datasets also showed that the transcriptional level of WEE1 was unaltered in atherosclerotic aortas (Figure S1A–E, Supporting Information). Considering that the phosphorylating change of WEE1 appears bigger than that of SGK1 (Figure 1B,C) and SGK1 has been reported to play a pivotal role in the development of atherosclerosis,^[^ ^16^ ^]^ we selected WEE1 for further investigation. We also examined the levels of p‐WEE1/WEE1 in human atherosclerotic carotid arteries. As shown in Figure 1D–G and p‐WEE1 were significantly up‐regulated during human atherogenesis, without the increase in WEE1 protein. These data indicated that the kinase activity, rather than the protein expression, of WEE1 may be involved in atherosclerosis.

We then examined the phosphorylating site and cellular distribution of p‐WEE1 in atherosclerotic aortas. WEE1 phosphorylation can occur at Ser‐642 (S642), Ser‐53 (S53), and Ser‐139 (S139). Only S642 phosphorylation of WEE1 has been reported to drive WEE1 activation,^[^ ^17^ ^]^ while S53 or S139 phosphorylation of WEE1 promotes its ubiquitin‐mediated degradation.^[^ ^18^ ^]^ We used the commercially available antibodies of p‐WEE1 (S53 and S139) and showed that HFD did not induce WEE1 phosphorylation at S53 and S139 in the aortas of HFD‐fed ApoE^−/−^ mice (Supplementary Figure S2A,B, Supporting Information). Thus, the in vivo data suggest that WEE1 phosphorylation at S642 is involved in atherosclerosis. Regarding the cellular distribution, the immunofluorescence staining showed that p‐WEE1^S642^ colocalized with F4/80, a macrophage marker, rather than with α‐SMA, a smooth muscle cell marker, in mouse aortas (Figure 1H,I). Figure 1H also showed that the level of macrophage p‐WEE1 was significantly increased in atherosclerotic plaques of HFD‐fed ApoE^−/−^ mice. We also analyzed the protein levels of WEE1 in mouse aortic vascular smooth muscle cells (MOVAS), human umbilical vein endothelial cells (HUVEC), and mouse primary peritoneal macrophages (MPMs), respectively. As shown in Figure 1J, the basal protein level of WEE1 in MPM was much higher than that in smooth muscle cells and endothelial cells. We next determined that p‐WEE1^S642^ level was increased in oxLDL‐challenged MPMs in a time‐dependent manner (Figure 1K), while oxLDL could not induce WEE1 phosphorylation in cultured MOVAS and HUVEC (Supplementary Figure S2C,D, Supporting Information). We also constructed plasmids containing wild‐type WEE1 (WEE1^WT^) or self‐inactivating mutation (WEE1^S642A^) and transfected these plasmids into the basal WEE1‐deficient MPMs. We detected the phosphorylated WEE1 using anti‐Pan Phospho‐Serine/Threonine antibody (Pan p‐S/T) in MPMs expressing WEE1^WT^, but not in MPMs expressing WEE1^S642A^ (Figure S2E, Supporting Information), further validating S642 as the only phosphorylating site. These data suggest that macrophage WEE1 is phosphorylated at S642 in mouse atherosclerotic lesions and the WEE1 activation may be involved in the progression of atherosclerosis.

### 2.2 WEE1 Activation Promotes oxLDL‐Induced Inflammatory Response in Macrophages

**Figure 2:**
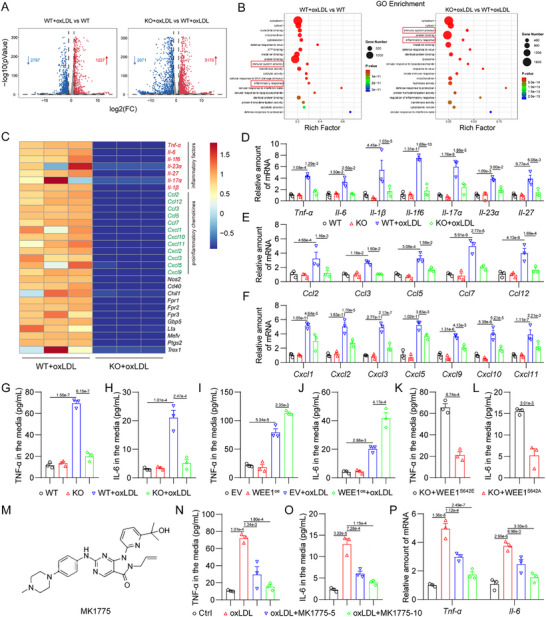
WEE1 activation mediates oxLDL‐induced inflammatory response in macrophages. A) MPMs isolated from WEE1^f/f^ and WEE1^MCKO^ mice were challenged with oxLDL (50 µg mL^−1^) for 6 h. Total RNA was sequenced to identify differentially expressed genes (DEGs). Volcano plot analysis of DEGs up‐regulated (red) and down‐regulated (blue) in WT+oxLDL group compared to WT group or in KO+oxLDL group compared to WT+oxLDL group. FC, fold change. B) GO enrichments of DEGs in WT+oxLDL group compared to WT group or in KO+oxLDL group compared to WT+oxLDL group. C) Heat map of TOP30 DEGs associated with inflammatory response in KO+oxLDL group compared to WT+oxLDL group. D–F) MPMs isolated from WEE1^f/f^ and WEE1^MCKO^ mice were challenged with oxLDL (50 µg mL^−1^) for 6 h. mRNA levels of inflammatory factors D) and proinflammatory chemokines E,F) were determined via RT‐qPCR (*n* = 3). The values were normalized to *Actb*. G,H) MPMs isolated from WEE1^f/f^ and WEE1^MCKO^ mice were challenged with oxLDL (50 µg mL^−1^) for 24 h. Protein levels of TNF‐α G) and IL‐6 H) were analyzed using ELISA (*n* = 3). I,J) MPMs transfected with WEE1 or empty vector (EV) were challenged with oxLDL (50 µg mL^−1^) for 24 h. Protein levels of TNF‐α I) and IL‐6 J) were analyzed using ELISA (*n* = 3). K,L) MPMs isolated from WEE1^MCKO^ mice were transfected with WEE1^S642E^ or WEE1^S642A^ for 24 h. Protein levels of TNF‐α K) and IL‐6 L) were analyzed using ELISA (*n* = 3). M) The chemical structure of MK1775. N.O) MPMs were pretreated with MK1775 (5 and 10 µm) or vehicle (DMSO, 1‰) for 1 h, followed by exposure of oxLDL (50 µg mL^−1^) for 24 h. Protein levels of TNF‐α N) and IL‐6 O) were analyzed using ELISA (*n* = 3). P) MPMs were pretreated with MK1775 (5 and 10 µm) or vehicle (DMSO, 1‰) for 1 h, followed by exposure of oxLDL (50 µg mL^−1^) for 6 h. mRNA levels of *Tnf‐α* and *Il‐6* were determined via RT‐qPCR (*n* = 3). The values were normalized to *Actb*. Data were shown as mean ± SEM. D–F and P, two‐way ANOVA followed by Tukey's test; G‐J and N,O, one‐way ANOVA followed by Tukey's test; K,L, Student's *t*‐test.

To investigate the role of macrophage WEE1 in atherosclerosis, we utilized transcriptome sequencing in oxLDL‐stimulated MPMs from the macrophage‐specific WEE1 knockout (WEE1^MCKO^) and littermate WEE1^f/f^ mice (**Figure** **2**A; Figure S3, Supporting Information). GO enrichment revealed that the effect of WEE1 deletion in oxLDL‐challenged macrophages may be related to “inflammatory response” and “immune system process” (Figure 2B), which were enriched in both comparisons, “WT+oxLDL versus WT” and “KO+oxLDL versus WT+oxLDL”. Macrophages are central to the pathogenesis and progression of atherosclerosis due to their capacity to initiate and sustain inflammatory and immune responses.^[^ ^19^ ^]^ In particular, the macrophage‐mediated activation of these responses constitutes a critical mechanism in the evolution of atherosclerotic lesions.^[^ ^20^ ^]^ Therefore, we selected the “inflammatory response” term in GO analysis for further investigation and considered that WEE1 knockout may affect oxLDL‐induced inflammation and immune response in macrophages. We screened out the top 30 differentially expressed genes (DEGs) associated with inflammation, including inflammatory factors and proinflammatory chemokines (Figure 2C; Table S1, Supporting Information). We then validated these genes by PCR analysis. The results showed that WEE1 knockout suppressed oxLDL‐induced upregulation of inflammatory factors and proinflammatory chemokines in MPMs (Figure 2D–F). We also confirmed that WEE1 deletion decreased the protein levels of TNF‐α and IL‐6 in MPMs challenged with oxLDL (Figure 2G,H). Besides oxLDL stimulation, we harvested the HFD‐fed mouse serum including hyperlipidemia to treat MPMs and found that WEE1 deletion inhibited hyperlipidemia‐induced TNF‐α and IL‐6 levels in cultured MPMs (Figure S4, Supporting Information). In contrast, WEE1 overexpression, accompanied by increased p‐WEE1 (Figure S5A, Supporting Information), exacerbated TNF‐α and IL‐6 releases in oxLDL‐challenged MPMs (Figure 2I,J).

Next, we examined if the pro‐inflammatory action of WEE1 attributed to its kinase activity, rather than its protein level. We constructed plasmids containing self‐activating (WEE1^S642E^) and self‐inactivating (WEE1^S642A^) mutations of WEE1 and transfected these plasmids into MPMs equally (Figure S5B, Supporting Information). As shown in Figure 2K,L, self‐activation of WEE1 facilitated the expression of TNF‐α and IL‐6 in MPMs without oxLDL stimulation, whereas WEE1 inactivation failed. Moreover, we used a selective small‐molecule inhibitor of WEE1 kinase activity, MK1775 (Figure 2M),^[^ ^21^ ^]^ which showed remarkable inhibition against WEE1 phosphorylation in MPMs at the doses of 5 and 10 µm (Figure S5C, Supporting Information). As expected, MK1775 treatment dose‐dependently inhibited oxLDL‐induced upregulation of TNF‐α and IL‐6 at both mRNA and protein levels in MPMs (Figure 2N–P). In conclusion, these data indicate that Ser‐642 phosphorylation of WEE1 followed by its activation mediates oxLDL‐induced inflammatory response in macrophages.

### 2.3 Macrophage‐Specific WEE1 Deletion Reduces Atherosclerotic Plaques in HFD‐Fed ApoE^−/−^ Mice

**Figure 3:**
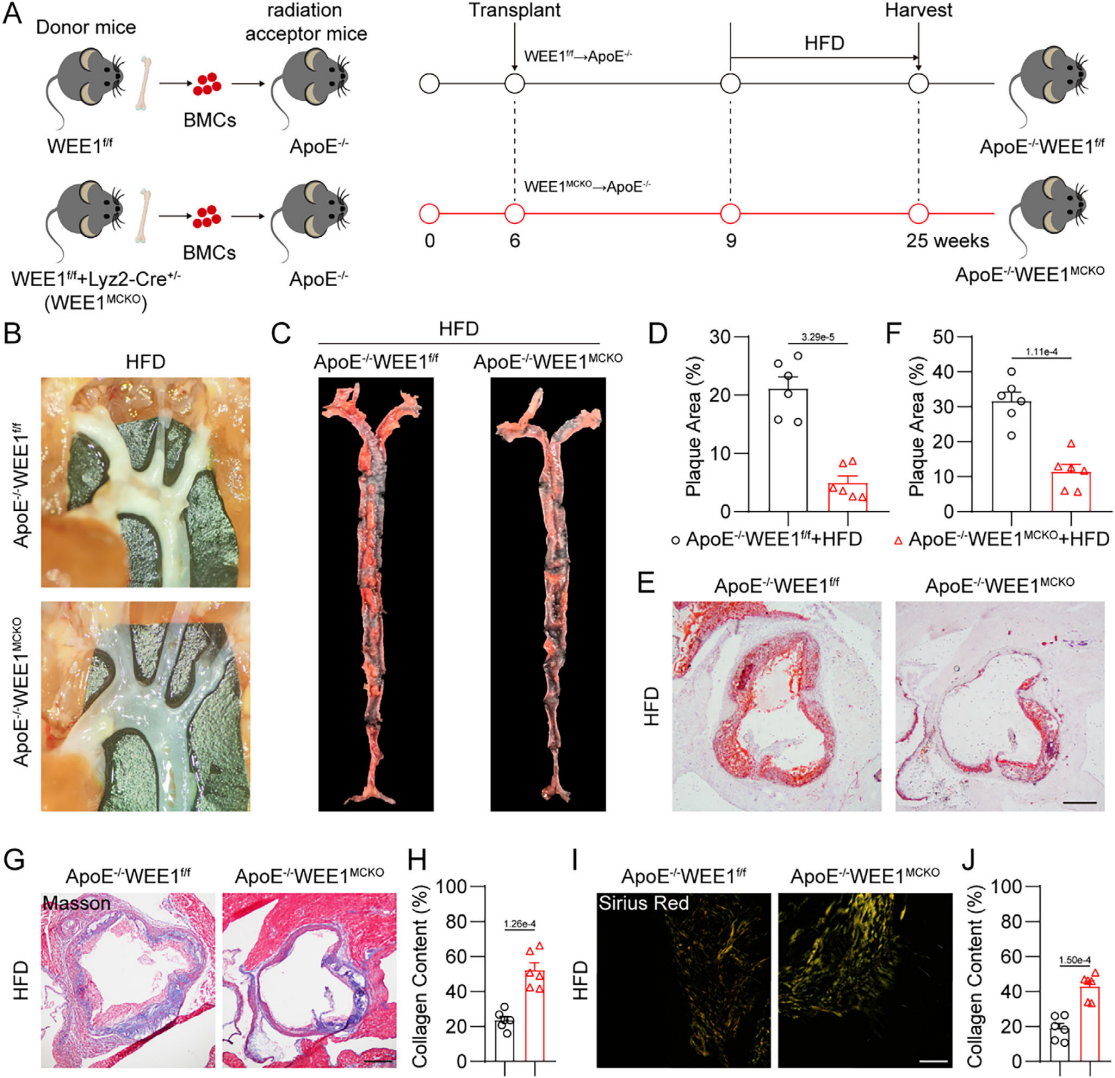
Macrophage‐specific WEE1 deletion reduces atherosclerotic plaques in HFD‐fed ApoE^−/−^ mice. A) Schematic diagram of the bone marrow transplantation and atherosclerosis model in mice. ApoE^−/−^ mice were irradiated and administered bone marrow cells (BMCs) from either WEE1^f/f^ or WEE1^MCKO^ mice. After 3 weeks, these mice were fed a high‐fat diet (HFD) for 16 weeks. B) Representative images of plaque lesion in aortic arches. C,D) Representative whole‐aorta Oil Red O staining C) and quantification D) of Oil Red O‐positive plaque lesion area in aortas. Plaque area was defined as percentage of total surface area of the aorta (*n* = 6). E,F) Representative images of Oil Red O staining E) and quantification F) of atherosclerotic lesion in aortic roots (scale bar = 250 µm, *n* = 6). G,H) Representative images of Masson's Trichrome staining G) and quantification H) for collagen deposition in aortic roots (Scale bar = 250 µm, *n* = 6). I,J) Representative images of Sirius Red staining I) and quantification J) for collagen deposition in aortic roots (Scale bar = 50 µm, *n* = 6). Data were shown as mean ± SEM. D, F, H, and J, Student's *t*‐test. For panels E, G, and I, plaques were analyzed from first appearance of intact three sinus valves.

WEE1^MCKO^ mice, as well as the littermate WEE1^f/f^ mice, were constructed to investigate the role of macrophage WEE1 in atherosclerosis. We first performed flow cytometry analysis on the myeloid progenitor subsets in the bone marrow cells and peripheral blood cells of WEE1^f/f^ and WEE1^MCKO^ mice, respectively. Our results demonstrated that macrophage‐specific WEE1 deletion did not affect the composition of myeloid progenitor subsets (Figures S6A–D and S7A–D, Supporting Information), indicating that WEE1 does not regulate the differentiation of myeloid cells. Then, ApoE^−/−^ mice were irradiated and administrated with bone marrow cells from WEE1^f/f^ or WEE1^MCKO^ mice to generate the ApoE^−/−^ mice with wide‐type WEE1 expression (ApoE^−/−^WEE1^f/f^) and the ApoE^−/−^ mice with macrophage‐specific WEE1 deletion (ApoE^−/−^WEE1^MCKO^), respectively (**Figure** **3**A; Figure S8A, Supporting Information). We first confirmed that macrophage‐specific WEE1 deletion did not affect the viability, normal vascular development, and serum lipid profile in LFD‐fed ApoE^−/−^ mice (Figure S8B–H, Supporting Information). Macrophage‐specific WEE1 deletion did not affect the body weight and serum lipid profile in HFD‐fed ApoE^−/−^ mice (Figure S9A–D, Supporting Information). As expected, HFD feeding led to the formation of atherosclerotic plaques in the aortas of ApoE^−/−^WEE1^f/f^ mice, whereas macrophage‐specific WEE1 deletion substantially diminished plaque size in the aortas of HFD‐fed ApoE^−/−^WEE1^MCKO^ mice (Figure 3B–D). Oil Red O staining of aortic roots revealed that macrophage WEE1 deletion mitigated atherosclerotic lesions in HFD‐fed ApoE^−/−^ mice (Figure 3E,F). Masson's trichrome and Sirius Red staining also showed more collagen content in atherosclerotic plaques in the ApoE^−/−^WEE1^MCKO^ mice, indicating that macrophage WEE1 deletion increased plaque stability (Figure 3G–J). Collectively, these findings suggest that macrophage‐specific WEE1 deletion alleviates HFD‐induced atherosclerotic progression without affecting serum lipid profile.

### 2.4 Macrophage‐Specific Wee1 Deletion Alleviates Inflammatory Cell Infiltration and Inflammation in Atherosclerotic Lesions

**Figure 4:**
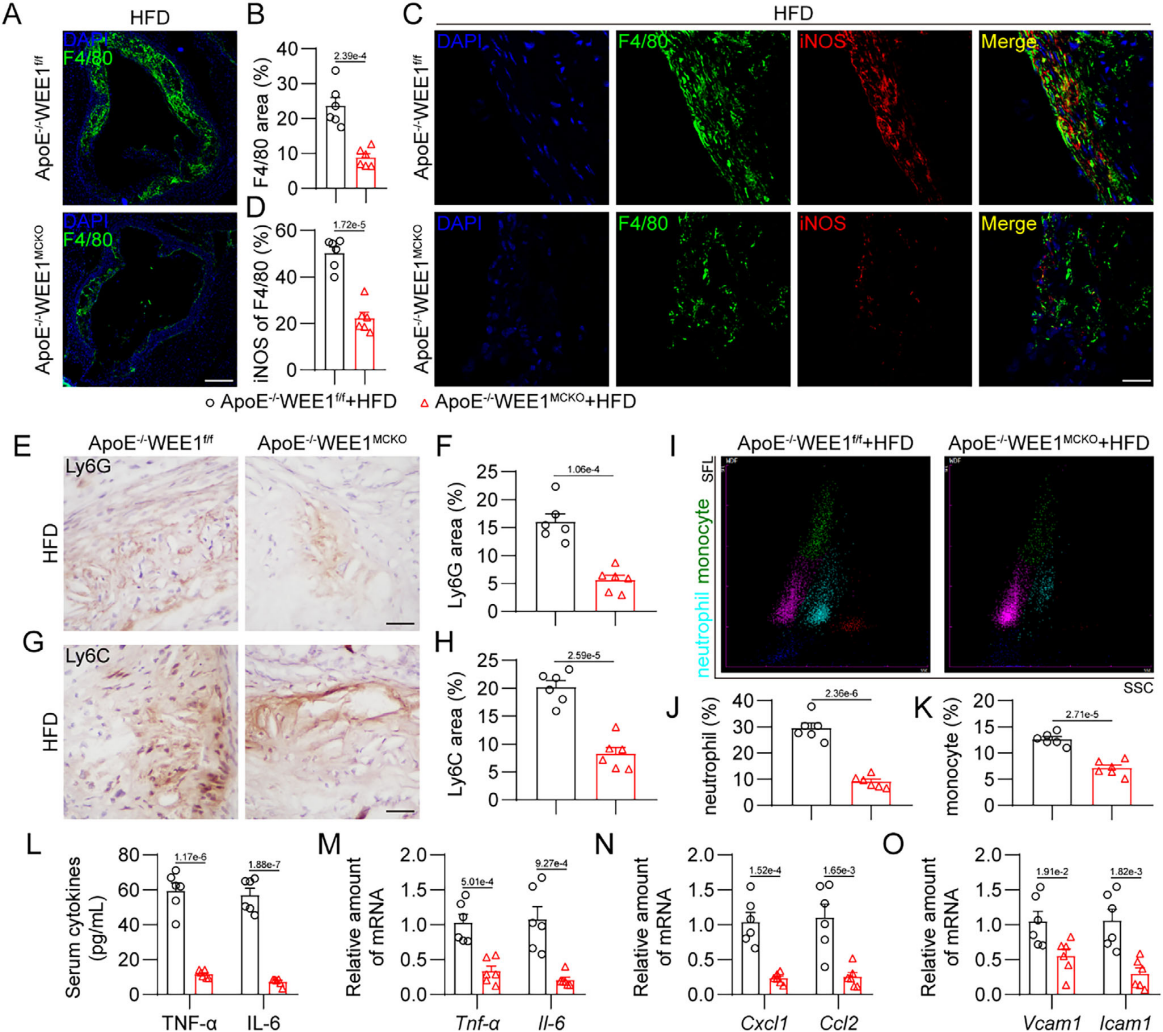
Macrophage‐specific WEE1 deletion alleviates inflammatory cell infiltration and inflammation in atherosclerotic lesions. A,B) Representative immunofluorescence staining images A) and quantification B) of F4/80 (green) in aortic roots. Tissues were counterstained with DAPI (blue). Scale bar = 250 µm, *n* = 6. C,D) Representative immunofluorescence staining images C) and quantification D) of F4/80 (green) and iNOS (red) in aortic roots (Scale bar = 50 µm, *n* = 6). Tissues were counterstained with DAPI (blue). E–H) Representative immunohistochemistry staining images and quantification of Ly6G E, F) and Ly6C G, H) in aortic roots (Scale bar = 25 µm, *n* = 6). I–K) Scatter diagram I) and quantification J–L) of neutrophils and monocytes in plasma measured by an automated blood cell analyzer (*n* = 6). SSC, side scatter; SFL, side fluorescence. L,M) Protein L) and mRNA M) levels of inflammatory cytokines TNF‐α and IL‐6 in serum or aortas. The values of mRNA levels were normalized to *Rn18s* (*n* = 6). N,O) mRNA levels of proinflammatory chemokines N) and adhesion molecules O) in aortas (*n* = 6). The values were normalized to *Rn18s*. Data were shown as mean ± SEM. B, D, F, H, J, K, and L‐O, Student's *t*‐test. For panels A, C, and E, plaques were analyzed from first appearance of intact three sinus valves.

Subsequently, we examined the inflammatory indexes in these mice. Immunofluorescence staining revealed a significant reduction in the infiltration of F4/80‐positive macrophages into atherosclerotic lesions in HFD‐fed ApoE^−/−^WEE1^MCKO^ mice, compared to that in ApoE^−/−^WEE1^f/f^ mice (**Figure** **4**A,B). We also observed a decrease in the level of iNOS‐positive pro‐inflammatory macrophages within atherosclerotic lesions of HFD‐fed ApoE^−/−^WEE1^MCKO^ mice (Figure 4C,D). Immunohistochemical analysis demonstrated that WEE1 deletion notably restrained the recruitment of Ly6G‐positive neutrophils (Figure 4E,F; Figure S10A, Supporting Information) and Ly6C‐positive monocytes (Figure 4G,H; Figure S10B, Supporting Information) into atherosclerotic plaques in HFD‐fed ApoE^−/‐^ mice, consistent with the quantitative assessment of neutrophils and monocytes in the plasma (Figure 4I–K). Furthermore, macrophage‐specific WEE1 deletion decreased the levels of inflammatory factors TNF‐α and IL‐6 in serum or aortas in HFD‐fed ApoE^−/−^ mice (Figure 4L,M). Similar changes were observed in the mRNA levels of proinflammatory chemokines (*Cxcl1*, *Ccl2*) and adhesion molecules (*Vcam1*, *Icam1*) in mouse atherosclerotic aortas (Figure 4N,O). Taken together, these data indicate that macrophage‐specific WEE1 deletion suppresses inflammatory cell infiltration and attenuates inflammatory response in atherosclerotic lesions.

### 2.5 Pharmacological Inhibitor of WEE1 Kinase Activity Prevents Atherosclerotic Progression and Inflammation in HFD‐Fed ApoE^−/−^ Mice

**Figure 5:**
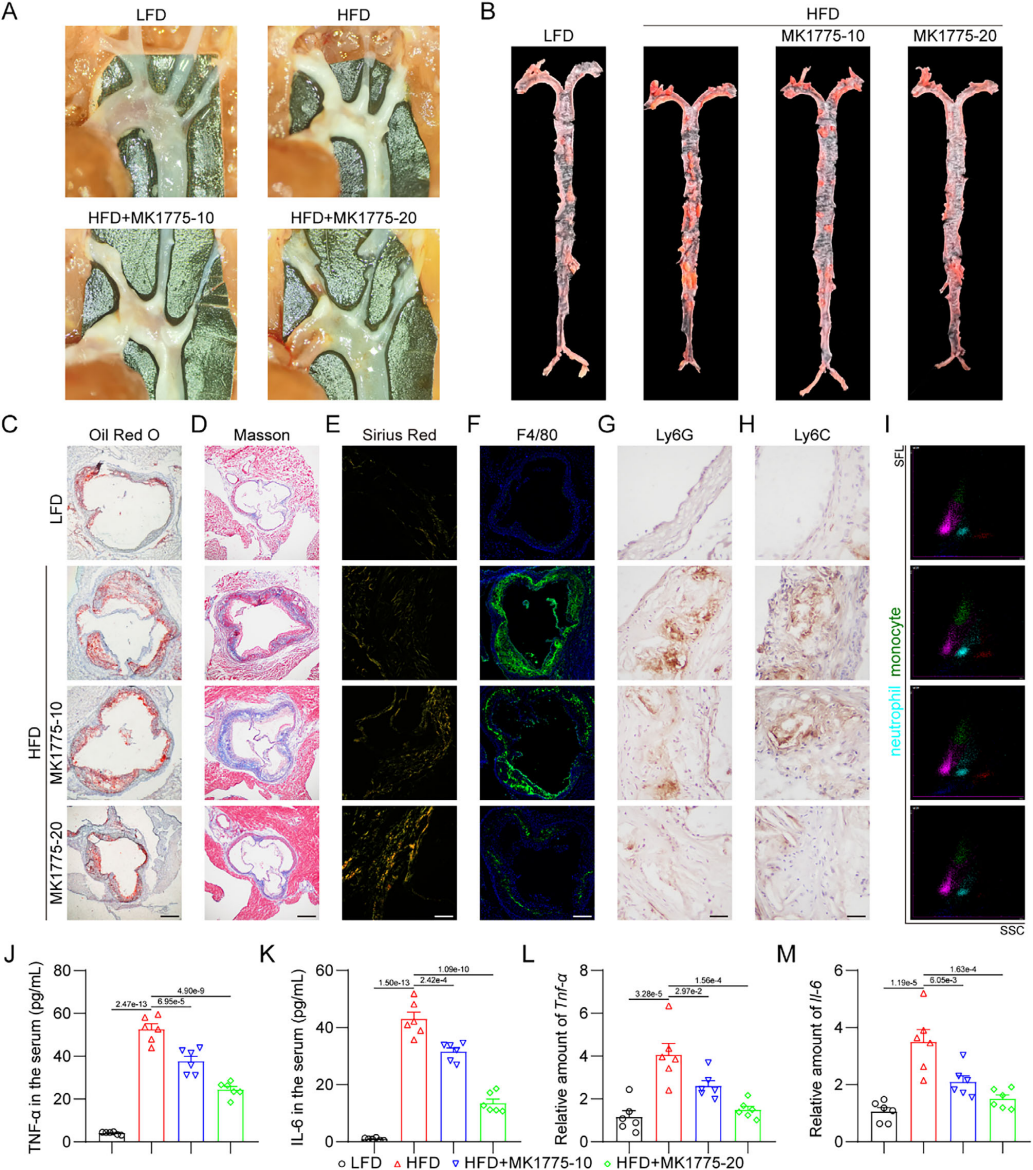
Pharmacological inhibitor of WEE1 kinase activity prevents atherosclerotic progression and inflammation in HFD‐fed ApoE^−/−^ mice. A) Representative images of plaque lesion in aortic arches. B) Representative whole‐aorta Oil Red O staining. C) Representative images of Oil Red O staining of atherosclerotic lesion in aortic roots (Scale bar = 250 µm). D) Representative images of Masson's Trichrome staining in aortic roots (Scale bar = 250 µm). E) Representative images of Sirius Red staining in aortic roots (Scale bar = 50 µm). F) Representative immunofluorescence staining images of F4/80 (green) in aortic roots. Tissues were counterstained with DAPI (blue). Scale bar = 250 µm. G,H) Representative immunohistochemistry staining images of Ly6G G) and Ly6C H) in aortic roots (Scale bar = 25 µm). I) Scatter diagram of neutrophils and monocytes in plasma measured by an automated blood cell analyzer. SSC, side scatter; SFL, side fluorescence. J–M) Protein J,K) and mRNA L,M) levels of inflammatory cytokines TNF‐α and IL‐6 in serum or aortas. The values of mRNA levels were normalized to *Rn18s* (*n* = 6). Data were shown as mean ± SEM. J–M, one‐way ANOVA followed by Tukey's test.

MK1775 was further employed in in vivo experiments to bolster translational significance. The dosages of MK1775 at 10 and 20 mg kg^−1^ per 2 days were determined based on previous studies.^[^ ^21^ ^]^ As illustrated in Supplementary Figure S11A–D (Supporting Information), MK1775 treatment had no discernible effects on the body weight and serum lipid profile in HFD‐fed ApoE^−/−^ mice. However, MK1775 notably reduced the plaque area in the aortas of HFD‐fed ApoE^−/−^ mice (**Figure** **5**A,B; Figure S12A, Supporting Information). Oil Red O staining of the aortic roots also demonstrated that MK1775 attenuated HFD‐induced atherosclerotic lesions (Figure 5C; Figure S12B, Supporting Information). Masson's trichrome and Sirius Red staining depicted increased collagen content in atherosclerotic plaques in MK1775‐treated groups (Figure 5D,E; Figure S12C,D, Supporting Information). Immunofluorescence and immunohistochemical staining further revealed that MK1775 significantly mitigated the infiltration of inflammatory cells (macrophages, neutrophils, and monocytes) into atherosclerotic lesions in HFD‐fed ApoE^−/−^ mice (Figure 5F–H; Figure S12E–G, Supporting Information), consistent with the quantitative analysis of neutrophils and monocytes in the plasma (Figure 5I; Figure S12H,I, Supporting Information). Furthermore, MK1775 diminished the levels of inflammatory factors (TNF‐α, IL‐6) in HFD‐fed ApoE^−/−^ mouse serum and aortas (Figure 5J–M). In summary, these findings validate that pharmacological inhibition of WEE1 kinase activity also alleviates atherosclerotic progression and inflammation in mice.

### 2.6 WEE1 Mediates Inflammatory Response via Phosphorylating p65 at S536 in Macrophages

**Figure 6:**
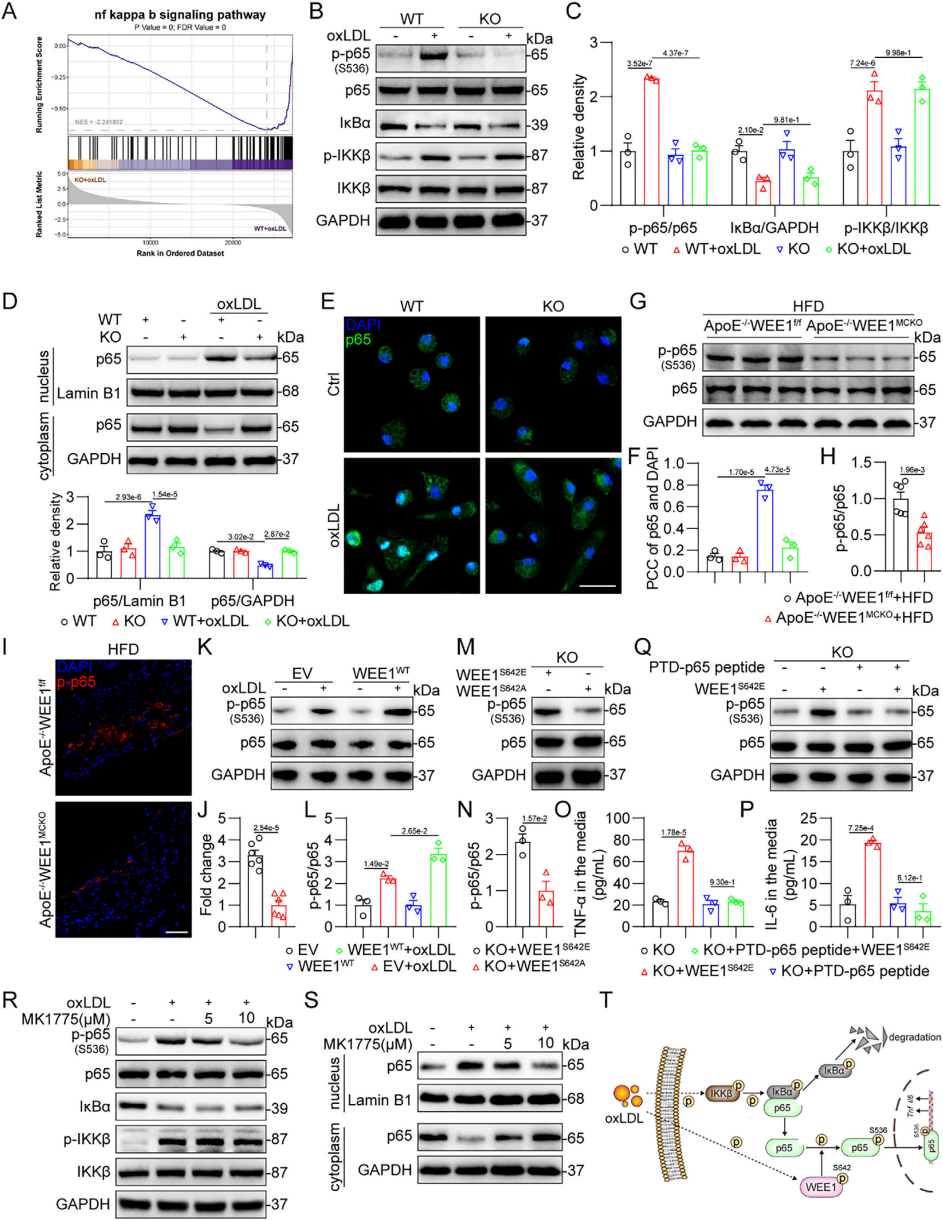
WEE1 mediates inflammatory response via phosphorylating p65 at S536 in macrophages. A) NF‐κB signaling pathway is enriched in gene‐set enrichment analysis (GSEA) of KO+oxLDL versus WT+oxLDL group. B,C) MPMs isolated from WEE1^f/f^ and WEE1^MCKO^ mice were challenged with oxLDL (50 µg mL^−1^) for 1 h. Western blot analysis B) and densitometric quantification C) of p‐p65, IκBα and p‐IKKβ. GAPDH, p65, and IKKβ were used as loading controls (*n* = 3). D) MPMs were treated as described in panel B). Western blot analysis and densitometric quantification of p65 in nucleus and cytoplasm. GAPDH was used as the loading control for cytosolic fractions. Lamin B1 was used as the loading control for nuclear fractions (*n* = 3). E,F) MPMs were treated as described in panel B). Representative immunofluorescence staining images E) and quantification F) of p65 (red) translocating into nucleus in MPMs. Cells were counterstained with DAPI (blue). Scale bar = 25 µm, *n* = 3. PCC, Pearson correlation coefficient. G,H) Western blot analysis G) and densitometric quantification H) of p‐p65 in aortic tissues of the mice. GAPDH and p65 were used as loading controls (*n* = 6). I,J) Representative immunofluorescence staining images I) and quantification J) of p‐p65 (red) in aortic roots. Tissues were counterstained with DAPI (blue). Scale bar = 50 µm, *n* = 6. Plaques were analyzed from first appearance of intact three sinus valves. K,L) MPMs transfected with WEE1 or EV were challenged with oxLDL (50 µg mL^−1^) for 1 h. Western blot analysis K) and densitometric quantification L) of p‐p65. GAPDH and p65 were used as loading controls (*n* = 3). M,N) MPMs isolated from WEE1^MCKO^ mice were transfected with WEE1^S642E^ or WEE1^S642A^ for 24 h. Western blot analysis M) and densitometric quantification N) of p‐p65. GAPDH and p65 were used as loading controls (*n* = 3). O,P) MPMs isolated from WEE1^MCKO^ mice were transfection with WEE1^S642E^ and then treated with PTD‐p65 peptide (100 µm) or vehicle (DMSO, 1‰) for 24 h. Protein levels of TNF‐α O) and IL‐6 P) were analyzed using ELISA (*n* = 3). Q) MPMs were treated as described in panel O,P). Western blot analysis of p‐p65. GAPDH and p65 were used as loading controls (*n* = 3). R) MPMs were pretreated with MK1775 (5 and 10 µm) or vehicle (DMSO, 1‰) for 1 h, followed by exposure of oxLDL (50 µg mL^−1^) for 1 h. Western blot analysis of p‐p65, IκBα, and p‐IKKβ. GAPDH, p65, and IKKβ were used as loading controls (*n* = 3). S) MPMs were treated as described in panel R). Western blot analysis and densitometric quantification of p65 in nucleus and cytoplasm. GAPDH was used as the loading control for cytosolic fractions. Lamin B1 was used as the loading control for nuclear fractions (*n* = 3). T) Working model of WEE1 modulating NF‐κB signaling pathway. Data were shown as mean ± SEM. C,D, two‐way ANOVA followed by Tukey's test; F, L, and O,P, one‐way ANOVA followed by Tukey's test; H, J, and N, Student's *t*‐test.

Next, we tried to explore how WEE1 mediates inflammatory response in macrophages. GSEA enrichment analysis of the RNA‐sequencing data revealed a potential association between the anti‐inflammatory effect of WEE1 deficiency and the NF‐κB signaling pathway (**Figure** **6**A). As we know, the transcriptional factor NF‐κB governs inflammatory gene expression and plays a pivotal role in atherosclerosis.^[^ ^22^ ^]^ In the activation of NF‐κB signaling cascade, the upstream IKKβ kinase was phosphorylated and then phosphorylated IκBα to promote IκBα degradation, which then released the NF‐κB p65 subunit. After phosphorylated, the free p65 subunit transfers from the cytosol to the nuclear to regulate target gene transcription. Therefore, we confirmed the effect of WEE1 on NF‐κB cascade. As shown in Figure 6B,C, WEE1 knockout suppressed p65 phosphorylation at S536, a key phosphorylation site for p65 activation,^[^ ^23^ ^]^ while WEE1 knockout did not affect IKKβ phosphorylation and IκBα degradation in oxLDL‐challenged macrophages. These data suggest that WEE1 may mediate p65 activation, independent of the classic upstream IKKβ and IκBα.

We then validated the regulation of WEE1 on p65 activation. Data in Figure 6D–F showed that WEE1 deletion reduced subsequent nuclear translocation of p65 in MPMs challenged with oxLDL. WEE1 deletion also inhibited p65 activation in MPMs challenged by mouse serum with hyperlipidemia (Figure S13A,B, Supporting Information). We also observed that macrophage‐specific WEE1 deletion suppressed p65 phosphorylation at S536 in atherosclerotic lesions of HFD‐fed ApoE^−/−^ mice (Figure 6G–J). In addition, WEE1 overexpression promoted p65 phosphorylation at S536 in MPMs challenged with oxLDL (Figure 6K‐L). We then determined that self‐activation mutation of WEE1 facilitated p65 phosphorylation in MPMs without oxLDL stimulation, whereas WEE1 inactivating mutation failed, indicating that the kinase activity of WEE1 is essential for promoting p65 phosphorylation in macrophages (Figure 6M,N).

Next, we explored whether p65 is indispensable for WEE1‐mediated NF‐κB activation and inflammatory gene expression. We transfected WEE1‐deficient MPMs with WEE1^S642E^ plasmid and then challenged the cells with a selective inhibitor of p65, PTD‐p65 peptide.^[^ ^24^ ^]^ Self‐activation of WEE1 induced p65 phosphorylation and inflammatory cytokine expression, while these changes were significantly reversed by PTD‐p65 peptide (Figure 6O,P; Figure S14A, Supporting Information). Besides, MK1775 treatment dose‐dependently inhibited oxLDL‐induced p65 phosphorylation at S536 and subsequent nuclear translocation, without the influences on IKKβ phosphorylation and IκBα degradation (Figure 6R,S; Figure S14B,C, Supporting Information). Consistently, MK1775 suppressed p65 phosphorylation at S536 in atherosclerotic lesions of HFD‐fed ApoE^−/−^ mice (Figure S15A–D, Supporting Information). These data validate that WEE1 kinase activity mediates inflammatory response in macrophages via phosphorylating p65 at S536 (Figure 6T).

### 2.7 WEE1 Directly Interacts with the RHD Domain of p65 via Kinase Domain to Phosphorylate p65 at S536

**Figure 7:**
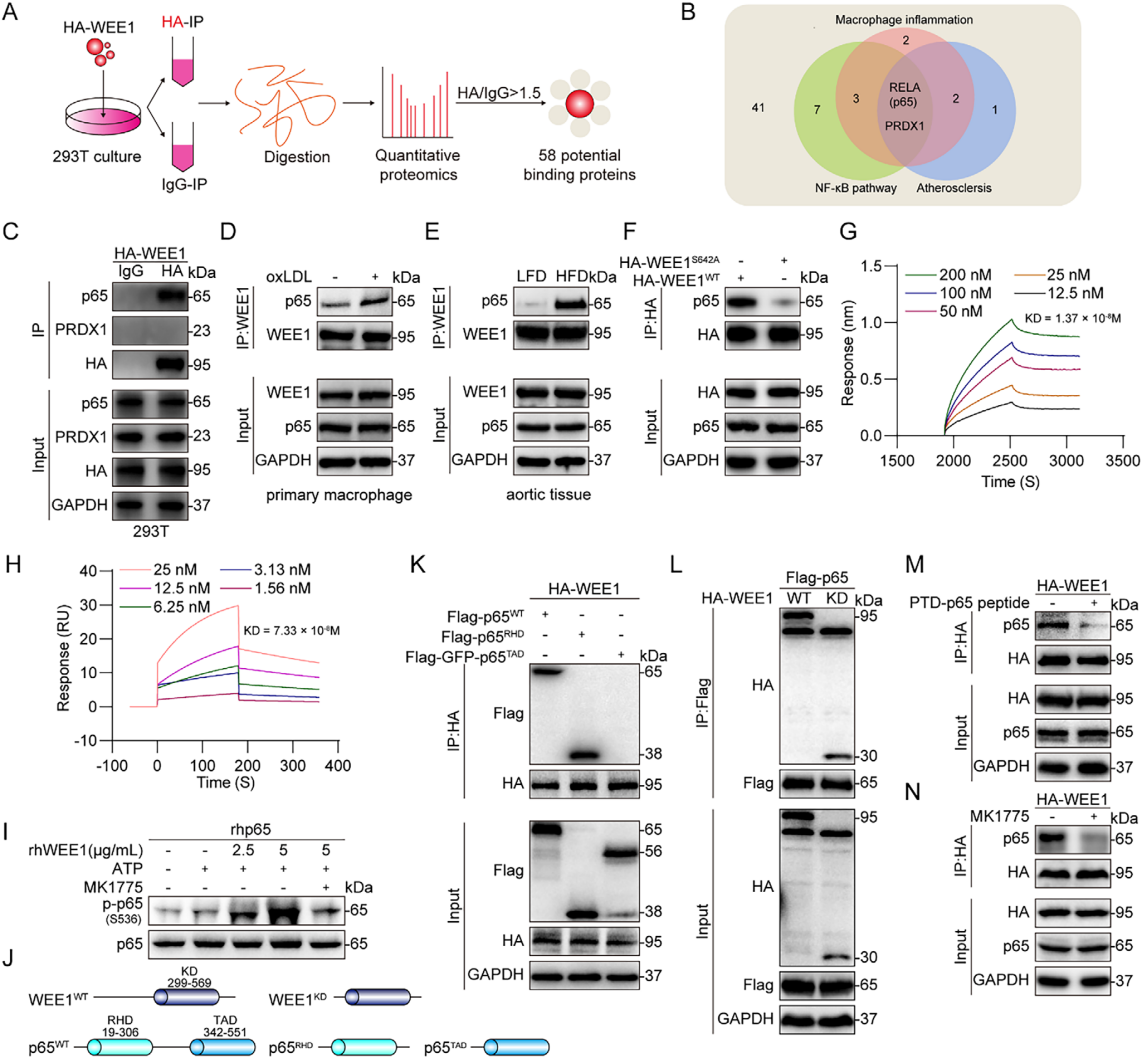
WEE1 directly interacts with the RHD domain of p65 via the kinase domain to phosphorylate p65 at S536. A) Schematic illustration of quantitative proteomic to identify proteins binding to WEE1. 293T cells were transfected with HA‐WEE1 plasmid for 24 h. B) Venn diagram representing potential WEE1‐binding proteins associated with macrophage inflammation, NF‐κB pathway, and atherosclerosis. C) Co‐immunoprecipitation of WEE1 with p65 and PRDX1 in 293T cells transfected with HA‐WEE1. HA‐WEE1 was immunoprecipitated by anti‐HA antibodies. IgG, immunoglobulin G. D) Co‐immunoprecipitation of WEE1 and p65 in MPMs challenged with oxLDL (50 µg mL^−1^) for 1 h. WEE1 was immunoprecipitated by anti‐WEE1 antibody. E) Co‐immunoprecipitation of WEE1 and p65 in aortic tissues of LFD and HFD‐fed ApoE^−/−^ mice. WEE1 was immunoprecipitated by anti‐WEE1 antibody. F) Co‐immunoprecipitation of WEE1 and p65 in 293T cells transfected with HA‐WEE1^WT^ or HA‐WEE1^S642A^. HA‐WEE1 or HA‐WEE1^S642A^ was immunoprecipitated by anti‐HA antibody. G) Bio‐layer interferometry (BLI) analysis showing direct interaction between WEE1 and p65. H) Surface plasmon resonance (SPR) analysis showing direct interaction between WEE1 and p65. I) rhWEE1 was incubated with rhp65 in the presence of ATP and MK1775(10 µm). rhp65 phosphorylation at S536 was detected by immunoblotting. J) The protein structure and the domain mutant constructs of WEE1 and p65. K) Co‐immunoprecipitation of WEE1 and p65 in 293T cells transfected with HA‐WEE1, Flag‐p65, and two p65 mutants. HA‐WEE1 was immunoprecipitated by anti‐HA antibodies. L) Co‐immunoprecipitation of WEE1 and p65 in 293T cells transfected with Flag‐p65, HA‐WEE1, or WEE1 mutant. Flag‐p65 was immunoprecipitated by anti‐Flag antibodies. M) Co‐immunoprecipitation of WEE1 and p65 in 293T cells. 293T cells were pretreated with PTD‐p65 peptide (100 µm) or vehicle (DMSO, 1‰) for 1 h followed by transfection with HA‐WEE1 for 24 h. HA‐WEE1 was immunoprecipitated by anti‐HA antibodies. N) Co‐immunoprecipitation of WEE1 and p65 in 293T cells. 293T cells were pretreated with MK1775 (10 µm) or vehicle (DMSO, 1‰) for 1 h followed by transfection with HA‐WEE1 for 24 h. HA‐WEE1 was immunoprecipitated by anti‐HA antibodies.

We tried to explore how WEE1 phosphorylates p65. We performed a quantitative proteomics analysis using cells transfected with HA‐WEE1 plasmid to identify WEE1‐binding proteins (**Figure** **7**A). The proteomics results revealed 58 potential WEE1‐binding proteins with HA/IgG ratio >1.5. Since WEE1 mediates inflammatory response via NF‐κB signaling pathway in oxLDL‐challenged macrophages and atherosclerosis, we checked the 58 WEE1‐binding proteins and found only 2 proteins have been reported to be associated with macrophage inflammation, NF‐κB pathway, and atherosclerosis: RELA (Transcription factor p65, p65)^[^ ^22^, ^23^ ^]^ and PRDX1^[^ ^25^ ^]^ (Figure 7B). We then confirmed the interaction between p65/PRDX1 and WEE1 in 293T cells by co‐immunoprecipitation assay. We found that WEE1 bound to p65, but showed no obvious interaction with PRDX1 (Figure 7C). Therefore, we selected p65 as the binding protein of WEE1 in macrophages. The second‐order mass spectrometry peptides of p65 in proteomics are shown in Figure S16 and Table S2 (Supporting Information).

The WEE1‐p65 complex was further found in both oxLDL‐challenged MPMs and atherosclerotic lesions of HFD‐fed ApoE^−/−^ mice (Figure 7D,E). In addition, we found that WEE1 inactivation by S642A mutation abolished WEE1 binding to p65 (Figure 7F), indicating that WEE1‐p65 complex formation is dependent on WEE1 activation. The direct interaction between rhWEE1 and rhp65 proteins was confirmed using bio‐layer interferometry (BLI) analysis, showing that rhWEE1 was bound to rhp65 with a KD value of 1.37 × 10^−8^ m (Figure 7G). Surface plasmon resonance (SPR) assay further validated the affinity, with a KD value of 7.33 × 10^−8^ m (Figure 7H). Importantly, we examined if rhWEE1 could directly catalyze rhp65 phosphorylation in the cell‐free system., rhWEE1 directly increased the phosphorylation of rhp65 at S536 and inhibiting WEE1 activity by MK1775 blocked rhWEE1‐mediated p65 phosphorylation (Figure 7I; Figure S17, Supporting Information).

We then investigated the detailed mode of WEE1‐p65 interaction using a series of constructed plasmids. WEE1 protein contains a kinase domain (KD)^[^ ^10^ ^]^ and p65 consists of two domains including RHD and TAD^[^ ^23^ ^]^ (Figure 7J). As shown in Figure 7K,L, WEE1 interacted with the RHD domain of p65 via its kinase domain. Given that PTD‐p65 peptide inhibits p65 phosphorylation by competitive binding to the RHD domain,^[^ ^24^ ^]^ we then confirmed that the interaction between WEE1 and the RHD domain of p65 was impeded by PTD‐p65 peptide in 293T cells transfected with HA‐WEE1 (Figure 7M). Besides, we found that blocking WEE1 activity by MK1775 also abolished WEE1‐p65 complex formation in 293T cells transfected with HA‐WEE1 (Figure 7N). These results show that WEE1 directly interacts with the RHD domain of p65 via its kinase domain to phosphorylate p65 at S536.

## 3 Discussion

Considering that PKs function by their kinase activity, in the present study, we utilized RNA‐seq‐based KEA to identify the activity‐changed PKs in mouse atherosclerotic aortas. Among five identified PKs (WEE1, SGK1, AURKB, ARAF, and PRKD3), we found that p‐WEE1 is up‐regulated in atherosclerotic lesions from both mice and humans, while the total level of WEE1 remains unchanged. We also found that WEE1 is mainly distributed in macrophages and its kinase activity mediates oxLDL‐induced inflammation in macrophages. We then evaluated the role of macrophage WEE1 in atherosclerosis both in vivo and in vitro. We showed that either macrophage‐specific deletion of WEE1 or pharmacological inhibition of WEE1 activity mitigates atherosclerotic development by reducing inflammatory responses in mice. Mechanistically, WEE1 activates NF‐κB and induces NF‐κB‐mediated inflammation in macrophages through directly binding to the RHD domain of p65 and phosphorylating p65 at S536. A schematic illustration of the main findings is presented in the Graphical Abstract.

Atherosclerosis is considered a chronic inflammatory disease. The macrophage inflammatory response intricately intertwines with atherosclerosis, spanning from its inception to the onset of complications.^[^ ^20b^ ^]^ Macrophages assume pivotal roles across all stages of atherosclerosis, from lesion initiation and expansion to necrosis culminating in plaque rupture and clinical manifestations of atherosclerosis.^[^ ^19^, ^26^ ^]^ Inflammatory mediators produced by macrophages control atheroma development. Consequently, pathological processes associated with macrophages emerge as significant targets for atherosclerosis therapy. Clinical trials reported that anti‐inflammatory IL‐1β antibody drugs, canakinumab,^[^ ^27^ ^]^ and colchicine^[^ ^28^ ^]^ could benefit patients during atherosclerotic treatment. Bao et al. determined that c‐type natriuretic peptide (CNP) alleviates macrophage inflammatory response in atherosclerosis.^[^ ^29^ ^]^ Here, we presented WEE1 as a new potential target for atherosclerosis treatment through regulating macrophage inflammation. Either macrophage‐specific WEE1 deletion or pharmacological inhibition significantly prevented atherosclerotic development in HFD‐fed ApoE^−/−^ mice by inhibiting inflammatory responses.

Known functions of WEE1 kinase are almost exclusively derived from studies in cancer diseases. WEE1‐specific small‐molecule inhibitor MK1775 has been applied to clinical practice to inhibit the progression of certain tumors.^[^ ^14^ ^]^ In this study, we found that WEE1 activation is involved in macrophage inflammation and atherosclerotic development. WEE1 kinase activity in macrophages, rather than the expression level, mediates oxLDL‐induced inflammatory responses via NF‐κB activation. We also demonstrated that self‐activation of WEE1 induces p65 phosphorylation and subsequent inflammatory gene expression in macrophages without oxLDL stimulation, whereas inactivating mutation of WEE1 abolishes those effects in macrophages. Thus, this is the first time to identify a new function of macrophage WEE1. One unanswered question arising from our study is how oxLDL/hyperlipidemia induces WEE1 phosphorylation or self‐phosphorylation in macrophages. It is possible that WEE1 phosphorylation and activation are induced by oncogenic mutation in cancers.^[^ ^30^ ^]^ Currently, no upstream kinases catalyzing WEE1 phosphorylation have been reported. Although the upstream mechanism for WEE1 activation in atherosclerosis needs to be further investigated, our findings point out new directions to broaden the clinical applications for WEE1 inhibitor MK1775 in cardiovascular diseases and even other inflammatory diseases. It is worth mentioning that MK1775 showed the gastrointestinal reaction side‐effect in its clinical trials treating cancers.^[^ ^31^ ^]^ Therefore, it is suggested to develop new small‐molecule inhibitors targeting the interaction between WEE1 and p65 to treat atherosclerosis, which may avoid the side effects of the classic anti‐cancer inhibitors of WEE1 kinase.

NF‐κB is a quintessential transcription factor that orchestrates the expression of a myriad of inflammatory genes in inflammation‐related diseases including atherosclerosis.^[^ ^22^ ^]^ Canonically, NF‐κB activity is regulated by the upstream IκBα and IKKβ.^[^ ^23^ ^]^ Activated IKKβ phosphorylates IκBα, leading to the degradation of p‐IκBα by ubiquitination. Subsequently, the NF‐κB p65 subunit, which is normally sequestered in the cytoplasm by the IκBα‐p65 complex, is released. After liberation, the free p65 subunit is phosphorylated and then translocated into the nucleus to regulate target gene transcription. In the whole cascade of NF‐κB activation, from IKKβ phosphorylation to p‐p65 nuclear translocation, it is unclear which PK directly phosphorylates the free p65 in atherosclerosis so far.^[^ ^32^ ^]^ Here, we found that WEE1 knockout or inactivation did not affect IKKβ phosphorylation and IκBα degradation, while blocked p65 phosphorylation and subsequent p‐p65 nuclear translocation in oxLDL‐challenged macrophages. We identified p65 as a substrate protein of WEE1 and showed that p‐WEE1 phosphorylated p65 via directly binding to it. Thus, our data support that WEE1 is a PK that phosphorylates the free p65 in macrophages, which provides new insight into the NF‐κB signaling cascade. It will be interesting to examine if WEE1 also phosphorylates the free p65 and if WEE1 is generally involved in NF‐κB signaling cascade in other inflammatory diseases.

We show that WEE1 directly interacts with the RHD domain of p65 via the kinase domain and phosphorylates p65 at S536. Although S536 is the most critical and classic phosphorylation site for p65 activation,^[^ ^23^ ^]^ some other serine/threonine residues of p65 have been recently reported to be phosphorylated. Wang et al. reported that casein kinase II phosphorylates p65 on Ser529.^[^ ^33^ ^]^ Mitogen‐ and stress‐activated protein kinase‐1 (MSK1) were reported to phosphorylate p65 at Ser276 in fibroblasts challenged with TNF‐α.^[^ ^34^ ^]^ Wang et al. demonstrated that HSV‐1 protein kinase US3 phosphorylates p65 at Ser75 to inhibit NF‐κB activation.^[^ ^35^ ^]^ Here, we did not confirm whether S536 is the only site phosphorylated by WEE1. It is imperative in future studies to utilize an inactivating mutant at S536 of p65 to rule out the possibility of other serine/threonine residues in the WEE1‐p65 pro‐inflammatory signal.

In conclusion, the current study identified up‐regulated WEE1 phosphorylation in macrophages in atherosclerotic lesions and showed that macrophage‐specific deletion or pharmacological inhibition of WEE1 attenuated atherosclerotic progression by inhibiting NF‐κB‐mediated inflammation both in vivo and in vitro. Interestingly, we identify WEE1 as a key element in NF‐κB signaling pathway and show that the activated WEE1 directly interacts with the RHD domain of p65 to phosphorylate p65 at S536. This finding provides new insight into WEE1 in mediating NF‐κB inflammatory cascade in macrophages and identifies WEE1 as a potential therapeutic target for atherosclerosis.

## 4 Experimental Section

### Reagents

Oxidized low‐density lipoproteins (oxLDL) were purchased from Yiyuan Biotechnology (Guangzhou, China). The endotoxin level of oxLDL was <0.5EU mg^−1^ (protein). Antibodies against GAPDH (#5174, 1:1000), p‐IKKβ (#2697, 1:1000), IKKβ (#8943, 1:1000), NF‐κB p65 (Transcription factor p65, #8242, 1:1000 for western blotting and 1:200 for immunofluorescence staining), p‐p65 (#3033, 1:1000 for western blotting and 1:200 for immunofluorescence staining), IκBα (#9242, 1:1000), WEE1 (#13 084, 1:1000) and p‐WEE1 (S642, #4910, 1:1000 for western blotting and 1:200 for immunofluorescence staining) were purchased from Cell Signaling Technology (Danvers, MA, USA). Antibodies against F4/80 (#ab6640, 1:200), α‐SMA (#ab7817, 1:200), iNOS (#ab178945, 1:200), and Lamin B1 (#ab133741, 1:1000) were purchased from Abcam (Cambridge, UK). Antibodies against Ly6G (#sc‐53515, 1:100) and Ly6C (#sc‐271811, 1:100) were purchased from Santa Cruz Biotechnology (Santa Cruz, CA, USA). Antibodies against Flag (#20543‐1‐AP and #66008‐4‐Ig, 1:1000) and HA (#51064‐2‐AP and #66006‐2‐Ig, 1:1000) were purchased from Proteintech (Wuhan, China). Antibodies against p‐WEE1 (S53, #bs‐5589R, 1:1000), p‐SGK1 (#bs‐3396R, 1:1000), p‐ARAF (#bs‐3421R, 1:1000) and p‐PRKD3 (#bs‐5564R, 1:1000) were purchased from Bioss (Beijing, China). Antibodies against Pan Phospho‐Serine/Threonine (#AP0893, 1:1000), p‐AURKB (#AP0948, 1:1000), SGK1 (#A1025, 1:1000), AURKB (#A19539, 1:1000), ARAF (#A16346, 1:1000) and PRKD3 (#A7084, 1:1000) were purchased from ABclonal (Wuhan, China). The Antibody against p‐WEE1 (S139, #TA7291, 1:1000) was purchased from Abmart (Shanghai, China). The Antibody against PRDX1 (#ET1702‐08, 1:1000) was purchased from Huabio (Hangzhou, China). Antibodies against Fixable Viability Stain 510 (#564 406, 1:100), APC‐CY7‐CD45 (#561 037, 1:100), FITC‐CD11b (#561 688, 1:100), APC‐LY6G (#560 599, 1:100), PE‐CY7‐CD11c (#561 022, 1:100), PERCP‐CY5.5‐MHC‐II (#562 363, 1:100) and CD16/CD32 (#553 141, 1:100) were purchased from BD Pharmingen (San Diego, USA). The Antibody against BV605‐LY6C (#128 035, 1:100) was purchased from Biolegend (San Diego, USA). MK1775 (#HY‐10993) and PTD‐p65 peptide (#HY‐P1832) were purchased from MedChemExpress (New Jersey, USA). Recombinant human WEE1 protein (rhWEE1) and recombinant human p65 (rhp65) were purchased from Abiocenter (Beijing, China).

### In Vivo Randomization and Blinding Procedures

Sample sizes were defined by a priori power calculation with G‐Power 3.1.9 software (http://www.gpower.hhu.de/), considering a statistical power of 80% and α = 0.05. The number of mice used in each animal experiment was six. A random number table was employed to perform randomization. Briefly, all animal experiments in the present study were performed and analyzed in a blinded manner. Treatment groups were assigned in a randomized fashion. Every mouse was assigned a temporary random number within the weight range. Mice were given their permanent numerical designation in the cages after they were randomly divided into each group. For each group, a cage was selected randomly from the pool of all cages. All data were collected and analyzed by two observers who were not aware of the group assignment or treatment of the mice.

### Human Atherosclerotic Samples

Human non‐atherosclerotic carotid artery tissues (NAS) or atherosclerotic carotid artery tissues (AS) were collected from three atherosclerotic patients who underwent carotid endarterectomy. The clinical parameters for the three human samples were listed in Table S3 (Supporting Information). Atherosclerotic carotid artery tissues were defined as a focal region of carotid intima‐media thickness (IMT) >1.5 mm and non‐atherosclerotic carotid artery tissues were defined as a focal region of carotid IMT <1.0 mm and acquired from regions adjacent to the atherosclerotic tissues, as reported previously.^[^ ^36^ ^]^ Each patient's lineal consanguinity provided written informed consent. All the experiments involving human samples were approved by the Ethics Committee of The First Affiliated Hospital of Wenzhou Medical University (Approval NO. 2023188) and conformed to the principles outlined in the Declaration of Helsinki.

### Animal Experiments

All animal care and experimental procedures were approved by the Wenzhou Medical University Animal Policy and Welfare Committee (approval ID: wydw2021‐0057). All animal experiments followed the Guide for the Care and Use of Laboratory Animals (National Institutes of Health, USA). Wildtype C57BL/6JGpt, ApoE knockout (ApoE^−/−^) mice on a C57BL/6JGpt background, C57BL/6JGpt‐Wee1^em1Cflox^/Gpt (WEE1^f/f^) mice, and C57BL/6JGpt‐Lyz2^em1Cin(iCre)^/Gpt (Lyz2‐Cre) mice were obtained from GemPharmatech (nanjing, China). Macrophage‐specific WEE1 knockout mice (WEE1^f/f^+Lyz2‐Cre^+/−^, WEE1^MCKO^) were generated with technical expertise from GemPharmatech. Animals were housed in a 12:12 h light‐dark cycle at a constant room temperature and fed a standard rodent diet. The animals were acclimatized to the laboratory for at least two weeks before initiating the studies. All animal experiments were performed and analyzed by blinded researchers.

For studies using WEE1 knockout mice, one week before transplantation, five‐week‐old male ApoE^−/−^ mice were put in a clean environment and given acidified water containing neomycin (1.1 mg L^−1^) and polymyxin B sulfate (1000 U L^−1^). Twelve hours prior to transplantation, mice were subjected to total body irradiation (6 Gy). For transplantation, ApoE^−/−^ mice were injected via tail vein (5 × 10^6^ bone marrow cells from pools of bone marrow from six‐week‐old male WEE1^f/f^ mice or WEE1^MCKO^ mice), as described previously.^[^ ^37^ ^]^ After three weeks, mouse primary peritoneal macrophages (MPMs) and tails of ApoE^−/−^WEE1^f/f^ and ApoE^−/−^WEE1^MCKO^ mice were harvested and validation of genotype in macrophages and tails was performed by PCR using the gene primers in Table S4 (Supporting Information). Thus, macrophage‐specific WEE1 knockout mice on an ApoE^−/−^ background (ApoE^−/−^WEE1^MCKO^) and ApoE^−/−^WEE1^f/f^ mice were generated. Then, ApoE^−/−^WEE1^f/f^ and ApoE^−/−^WEE1^MCKO^ mice were fed a low‐fat diet (LFD) containing 10 kcal.% fat, 20 kcal.% protein and 70 kcal.% carbohydrate (H10010, HFK Bioscience, Beijing, China) or a high‐fat diet (HFD) containing 40 kcal.% fat, 20 kcal.% protein, 40 kcal.% carbohydrate and 1.25% cholesterol (H10540, HFK Bioscience) for 16 weeks. The number of ApoE^−/−^WEE1^f/f^+HFD and ApoE^−/−^WEE1^MCKO^+HFD was 12 per group. Six mice were used for full‐length aortic Oil Red O staining and the remaining six mice were used for other experiments. Body weight was recorded weekly. All mice were sacrificed under sodium pentobarbital anesthesia, and blood and aortas samples were collected. Aortic roots were used for Oil Red O staining, Sirius Red staining, Masson's trichrome staining, and immunostaining. Thoracic and abdominal aortas were used for western blotting and real‐time quantitative PCR.

For studies involving WEE1 inhibitor MK1775, eight‐week‐old ApoE^−/−^ mice were randomly divided into four groups: (i) LFD group: mice fed an LFD and intragastrically treated with 1% CMC‐Na vehicle control; (ii) HFD group: mice fed an HFD and intragastrically treated with 1% CMC‐Na vehicle control; (iii) HFD+ MK1775‐10 group: mice fed an HFD and intragastrically treated with 10 mg kg^−1^/2d MK1775 reconstituted in 1% CMC‐Na solution; (iv) HFD+ MK1775‐20 group: mice fed a HFD and intragastrically treated with 20 mg k^−1^g/2d MK1775 reconstituted in 1% CMC‐Na solution. The MK1775 dose was selected based on previous studies.^[^ ^21^ ^]^ MK1775‐treated mice were first fed an LFD or HFD for 8 weeks, followed by MK1775 treatment for another 8 weeks. Body weight was recorded weekly. All mice were sacrificed under sodium pentobarbital anesthesia at week 16, and blood samples were collected. Aortas were fixed in 4% paraformaldehyde or snap‐frozen in liquid nitrogen. Aortic roots were used for Oil Red O staining, Sirius Red staining, Masson's trichrome staining, and immunostaining. Thoracic and abdominal aortas were used for western blotting and real‐time quantitative PCR.

### Atherosclerotic Lesion Analysis

Oil Red O staining of whole aortas and aortic roots was utilized to determine the extent of atherosclerosis, as reported previously.^[^ ^38^ ^]^ For whole aorta lesion analysis, the whole aorta and aortic sinus were dissected, opened longitudinally from the heart to the iliac arteries, and stained with Oil Red O (G1260, Solarbio, Beijing, China). The heart and proximal aorta were collected and embedded in optimum cutting temperature compound for quantification of plaque lesions. Serial 6 µm‐thick cryosections of the aortic sinus were obtained from each mouse. The sections were stained with Oil Red O and hematoxylin for plaque size analysis.

Cryosections were used for Ly6G and Ly6C immunohistochemistry, Sirius Red staining (G1472, Solarbio), and Masson's trichrome staining (G1340, Solarbio). For immunohistochemistry, slides were fixed in cold methanol and permeabilized using 0.5% Triton‐X. Sections were blocked with 3% H_2_O_2_ and then with 5% bovine serum albumin for 30 min. Primary Ly6G (1:200) and Ly6C (1:200) antibodies were added. Sections were incubated at 4 °C overnight. Horseradish peroxidase‐conjugated secondary antibodies and DAB were used for detection.

Frozen sections were used for immunofluorescence staining. Slides were fixed in cold methanol and permeabilized using 0.5% Triton‐X. Then, slides were blocked using 5% bovine serum albumin for 30 min and incubated overnight with primary antibodies. Alexa‐488 and Alexa‐647 conjugated secondary antibodies (Abcam, 1:200) were used for detection. Images were captured using a fluorescence microscope (Nikon, Tokyo, Japan).

### Analysis of Leukocytes in Plasma

Plasma neutrophils and monocytes were analyzed using an automated blood cell analyzer (XN‐1000, Sysmex, Kobe, Japan).

### Cytokine Measurements

The levels of TNF‐α and IL‐6 in the serum and cell culture media were determined using ELISA kits (Cat#. 88‐7324‐76 and 88‐7064‐76, Thermo Fisher, CA, USA).

### Cell Culture

Mouse primary peritoneal macrophages (MPMs) were isolated as described previously.^[^ ^39^ ^]^ Briefly, mice received a single intraperitoneal injection of 6% thioglycolate solution. Two days later, the mice were euthanized and the peritoneal cavity was flushed with RPMI‐1640 medium (Gibco, Eggenstein, Germany). Samples were centrifuged, and the cell suspension was plated in RPMI‐1640 medium containing 10% fetal bovine serum (FBS, Gibco) and 1% penicillin/streptomycin (Invitrogen, Waltham, MA, USA). Nonadherent cells were removed 2 h after seeding the cell suspension. 293T cells (GNHu17), mouse aortic vascular smooth muscle cells (MOVASs), human umbilical vein endothelial cells (HUVECs) were purchased from Shanghai Institute of Biochemistry and Cell Biology (Shanghai, China) and cultured in high‐glucose Dulbecco's modified Eagle's medium (DMEM; Gibco) with 10% FBS and 1% penicillin/streptomycin. All cells were cultured in a humidified incubator maintained at 37 °C and 5% CO_2_.

### Gene Overexpression

Gene overexpression in cells was achieved by transfecting specific plasmids. Plasmid encoding HA‐WEE1^WT^, HA‐WEE1^S642E^, HA‐WEE1^S642A^, HA‐WEE1^KD^, Flag‐p65^WT^, Flag‐p65^RHD,^ and Flag‐GFP‐p65^TAD^ were constructed by GENEWIZ (Suzhou, China). Transfection of MPMs with plasmids was performed using Lipofectamine 3000 (Thermo Fisher Scientific, CA, USA) and Nucleic Acid Transfection Enhancer (NATE, Invivogen, Toulouse, France) as described previously.^[^ ^40^ ^]^ Transfection of 293T cells with plasmids was performed using Lipofectamine 3000.

### Western Blotting and Co‐Immunoprecipitation

Total protein from cells and aortic tissues was extracted using a lysis buffer (AR0103, Boster Biological Technology, Pleasanton, CA, USA). Proteins were separated by 10% sodium dodecyl sulfate‐polyacrylamide gel electrophoresis (SDS‐PAGE) and transferred to polyvinylidene fluoride membranes. Before adding specific primary antibodies, the membranes were blocked in Tris‐buffered saline (pH 7.4, containing 0.1% Tween 20 and 5% non‐fat milk) for 1.5 h at room temperature. Protein bands were detected by incubation with horseradish peroxidase‐conjugated secondary antibodies and an enhanced chemiluminescence reagent (Bio‐Rad, Hercules, CA, USA). All immunoblot exposures were in the linear range and quantitative. Band densities were quantified using ImageJ software (version 1.38e, NIH, Bethesda, MD, USA) and normalized to the loading controls.

For co‐immunoprecipitation assays, cell extracts prepared following treatments were incubated with indicated antibodies at 4 °C overnight. Then the proteins were immunoprecipitated with Protein A+G Agarose (P2012, Beyotime, Shanghai, China) at 4 °C for 2 h. Immunoprecipitation samples were immunoblotted for co‐precipitated protein detection. Total lysates were subjected to western blot analysis as input controls. Protein interactions were quantified using ImageJ software.

### Real‐Time Quantitative PCR

Total RNA was extracted from cells or aortic tissues using RNAiso Plus (9109, Takara, Shiga, Japan). Reverse transcription was performed using the PrimeScript RT Reagent Kit with gDNA Eraser (RR047A, Takara, Shiga, Japan). Quantitative PCR was performed using TB Green Premix Ex Taq II (RR820A; Takara, Shiga, Japan) in a QuantStudio 3 Real‐Time PCR System (Thermo Fisher Scientific, Carlsbad, CA, USA). The efficiency of PCR amplification was required to be 90–110%. The CT values were normalized to *Rn18s* or *β‐actin* and the 2^−△△CT^ method was used to calculate the relative amount of target mRNA. The primers were obtained from Thermo Fisher Scientific. The primer sequences used in this study are listed in Table S5 (Supporting Information).

### Transcriptome Sequencing

MPMs isolated from WEE1^f/f^ and WEE1^MCKO^ mice were challenged with or without oxLDL (50 µg mL^−1^) for 6 h. Total RNA from the MPMs (*n* = 3 per group) was collected using RNAiso Plus and subjected to genome‐wide transcriptomic analysis using LC‐Bio Tech. LTD (Hangzhou, China). Differentially expressed genes (DEGs) were selected with fold change >2 or fold change <0.5 and *p*‐value < 0.05. Gene‐set enrichment analysis (GSEA, https://www.gsea‐msigdb.org/gsea/index.jsp) of the signaling pathways and GO enrichment analysis were performed among the total DEGs by LC‐Bio Tech. LTD (https://www.lc‐bio.cn/). The raw data was deposited in the GEO repository (GSE262427).

### Quantitative Proteomics

Anti‐HA antibody was added to the lysate of 293T cells transfected with HA‐WEE1 for co‐immunoprecipitation and IgG was used as a negative control to exclude non‐specific binding during co‐immunoprecipitation. 3D label‐free quantitative proteomics analysis was performed by PTM Bio (Hangzhou, China). For digestion, the protein solution was reduced with 5 mm dithiothreitol for 30 min at 56 °C and alkylated with 11 mm iodoacetamide for 15 min at room temperature in darkness. The protein sample was then diluted by adding 100 mM TEAB to urea concentration less than 2 m. Finally, trypsin was added at 1:50 trypsin‐to‐protein mass ratio for the first digestion overnight and 1:100 trypsin‐to‐protein mass ratio for a second 4 h‐digestion. Finally, the peptides were desalted by C18 SPE column. Peptides were separated using an EASY‐nLC 1200 UPLC system (ThermoFisher Scientific). The separated peptides were analyzed in Orbitrap Exploris 480 (ThermoFisher Scientific) with a nano‐electrospray ion source. The resulting MS/MS data were processed using MaxQuant search engine (v.1.6.15.0). Tandem mass spectra were searched against the human SwissProt database (20 422 entries) concatenated with reverse decoy database. The FDR at the peptide and protein level data was set at 1%. The substrate proteins that may bind to WEE1 according to the HA/IgG ratio (>1.5) in the proteomics data to exclude non‐specific binding during co‐immunoprecipitation were screened. The raw data was deposited in the PRIDE repository (PXD050932). The whole proteomic data (both peptide and protein level data) was uploaded for HA and IgG in the Supplementary Files‐Proteomic data.

### Kinase enrichment Analysis (KEA)

Publicly available mouse transcriptome data GSE21419,^[^ ^41^ ^]^ GSE213303,^[^ ^42^ ^]^ GSE163657^[^ ^43^ ^]^ and GSE205929^[^ ^44^ ^]^ were acquired from the Gene Expression Omnibus database (https://www.ncbi.nlm.nih.gov/geo/). In GSE21419 dataset, ApoE^−/−^ mice were maintained on a standard mouse chow and aortas were harvested at the age of 78 weeks and divided into plaque areas and normal areas. In GSE213303, LDLR^−/−^ male mice were fed a low cholesterol or high cholesterol diet for 9 weeks and aortas were acquired. In GSE163657, ApoE^−/−^ mice were fed a chow or western diet for 22 weeks and aortas were acquired. In GSE205929, LDLR^−/−^ mice were fed a chow or high‐fat diet for 12 weeks and aortas were acquired. The four public mouse datasets were further used for kinase enrichment analysis (KEA) at BioJupies (https://maayanlab.cloud/biojupies/). Kinase enrichment analysis (KEA) predicts activity‐changed protein kinases by analyzing the downstream differentially expressed genes.^[^ ^9^ ^]^ Enrichment results were generated by analyzing the up‐regulated and down‐regulated gene sets using Enrichr.^[^ ^45^ ^]^ The following library was used for the analysis: ARCHS4_Kinases_Coexp. Significant results are determined by using a cut‐off of adjusted *p*‐value < 0.05 after applying Benjamini‐Hochberg correction.

### Bio‐Layer Interferometry (BLI) Analysis

Binding kinetics measurements were performed using Bio‐Layer Interferometry on FortéBio Octet Red 96 instrument. Purified rhp65 was biotinylated using Biotinylation Kit (G‐MM‐IGT, GENEMORE) following the manufacturer's instructions. The biotin labeled‐p65 protein was loaded onto super streptavidin (SSA) sensors (18‐5057, Octet) with 200 µL 25 mm HEPES, 150 mm NaCl, and 0.02% (v/v) Tween‐20 as running buffer. After equilibrium, association and dissociation measurements were carried out using serial dilutions of rhWEE1 protein (12.5, 25, 50, 100, and 200 nm).

### Surface Plasmon Resonance (SPR) Analysis

The binding affinity of rhWEE1 to rhp65 proteins was determined using the Biacore T200 Protein Interaction Assay system (GE Healthcare, Pittsburgh, PA) with a CM7 sensor chip (29‐1470‐20). Purified rhWEE1 was dissolved in 10 mm acetate acid buffer (pH 5.0), and then an amine coupling kit (GE BR‐1000‐50, GE Healthcare) was used to immobilize rhWEE1 on the chip. Different concentrations of rhp65 were prepared with running buffer (PBS containing 5% DMSO). Sensor and sample plates were placed in the instrument. The interactions were determined at a flow rate of 30 µL·min^−1^ for 250 s during the association phase, followed by 250 s for the dissociation phase at 25 °C. The data were analyzed using Biacore T200 manager software (GE Healthcare). Binding kinetic parameters were calculated by global fitting of the kinetic data from various concentrations of rhp65 using a 1:1 Langmuir binding model.

### Cell‐Free WEE1 Kinase Activity Assay

To examine WEE1 kinase activity on p65, the level of rhp65 phosphorylation by rhWEE1 was determined. Briefly, 2.5 or 5 µg mL^−1^ rhWEE1 and 100 µg mL^−1^ rhp65 were mixed with 1× Kinase reaction buffer (50 mm HEPES (pH 7.5), 1 mm EGTA, 0.01 mm BSA, 0.01% Tween‐20, 10 mm MgCl_2_, 2 mm MnCl_2_, 2 mm DTT) in a 50‐µL reaction containing 100 µm ATP. Samples were incubated at 30 °C for 1 h and stopped by adding SDS loading buffer. Phosphorylated p65 protein was detected by immunoblotting.

### Flow Cytometry Assay

All cell suspensions were carefully washed and stained with corresponding antibodies for 30 min on ice. Flow cytometry analysis was performed using BD Fortessa (BD Biosciences, San Jose, CA) under appropriate fluorescence compensation. Myeloid cells subsets were gated using FlowJo software (TreeStar, San Carlos, CA). Dendritic cells were identified as CD45^+^MHC‐II^+^CD11c^+^ subsets, neutrophils as CD45^+^CD11b^+^Ly6G^+^ subsets, and monocytes as CD45^+^CD11b^+^Ly6C^+^ subsets.

### Statistical Analysis

All data were collected and analyzed by two observers who were not aware of the group assignment or treatment. Data represented at six biological replicates in animal experiments and three biological replicates in cell experiments and were expressed as mean ± standard error of the mean (SEM). The exact group size (n) for each experiment is provided and “n” refers to biological replicates, not technical replicates. For in‐vitro experiments, as each experimental data set is an average of many cultured cells, we assumed the data was normally distributed based on the central limit theorem. For the analysis of in‐vivo experiments, our sample sizes were six, and the normal distribution of each data was evaluated for normal distribution using the Shapiro‐Wilk test. *P* > 0.05 on test indicated that the data were approximately normally distributed for each group. Comparisons between two groups were analyzed using a two‐tailed unpaired Student's *t*‐test. One‐way ANOVA followed by Tukey's test was used to compare more than two data groups. For samples that required a single individual to be measured more than once, data sets were analyzed independently using two‐way repeated‐measures ANOVA analysis with a single pooled variance and a Tukey correction for pairwise comparisons within groups for each data set. Statistical significance was set at *p* < 0.05. Post‐tests were run only if F achieved *p* < 0.05 and there was no significant variance in homogeneity. Statistical analyses were performed using GraphPad Prism 8.0 software (GraphPad, San Diego, CA, USA).

## Conflict of Interest

The authors declare no conflict of interest.

## Author Contributions

Z.H. and S.S. contributed equally to this work. G.L., Y.W., and Z.H. contributed to the literature search and study design; G.L. and Z.H. participated in the drafting of the article; Z.H., S.S., W.L., M.W., Y.Y., J.M., and X.L. carried out the experiments; G.W. collected the Clinical samples; W.H., Y.W., and G.L. revised the manuscript; X.H., Z.X., W.L., and X.L. contributed to data collection and analysis.

## Supporting information



Supporting Information

## Data Availability

The data that support the findings of this study are available from the corresponding author upon reasonable request.
