# Supplementary material for: Macrophage WEE1 Directly Binds to and Phosphorylates NF‐κB p65 Subunit to Induce Inflammatory Response and Drive Atherosclerosis
Source: Adv Sci (Weinh). 2025 Apr 9;12(26):2503192. doi: 10.1002/advs.202503192 (PMC12245028; doi:10.1002/advs.202503192)
Supplement: Supplementary file 1 — Supporting Information [file ADVS-12-2503192-s001.docx]

*Supplemental Material*

**Macrophage WEE1 directly binds to and phosphorylates NF-κB p65 subunit to induce inflammatory response and drive atherosclerosis**

The Supplemental Material includes 17 figures and 5 tables.

**
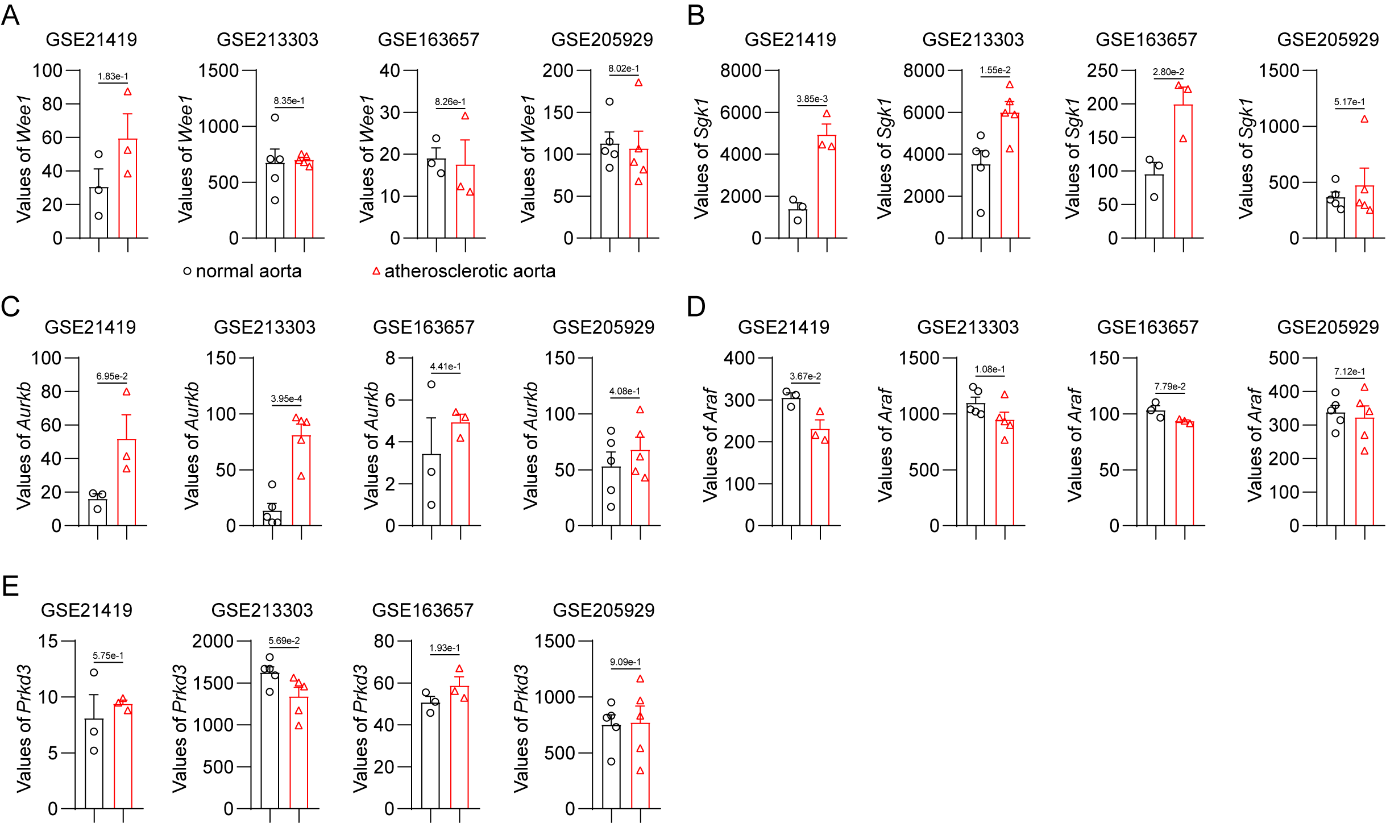
**

**Supplementary Figure S1.** **The mRNA levels of five protein kinases in all four datasets.** (A-E) Transcriptional level of *Wee1* (A), *Sgk1* (B), *Aurkb* (C), *Araf* (D) and *Prkd3* (E) in atherosclerotic aortas compared to normal aortas from 4 public mouse datasets (GSE21419, GSE213303, GSE163657 and GSE205929). Data were shown as mean ± SEM. A-E, Student’s t-test.

**
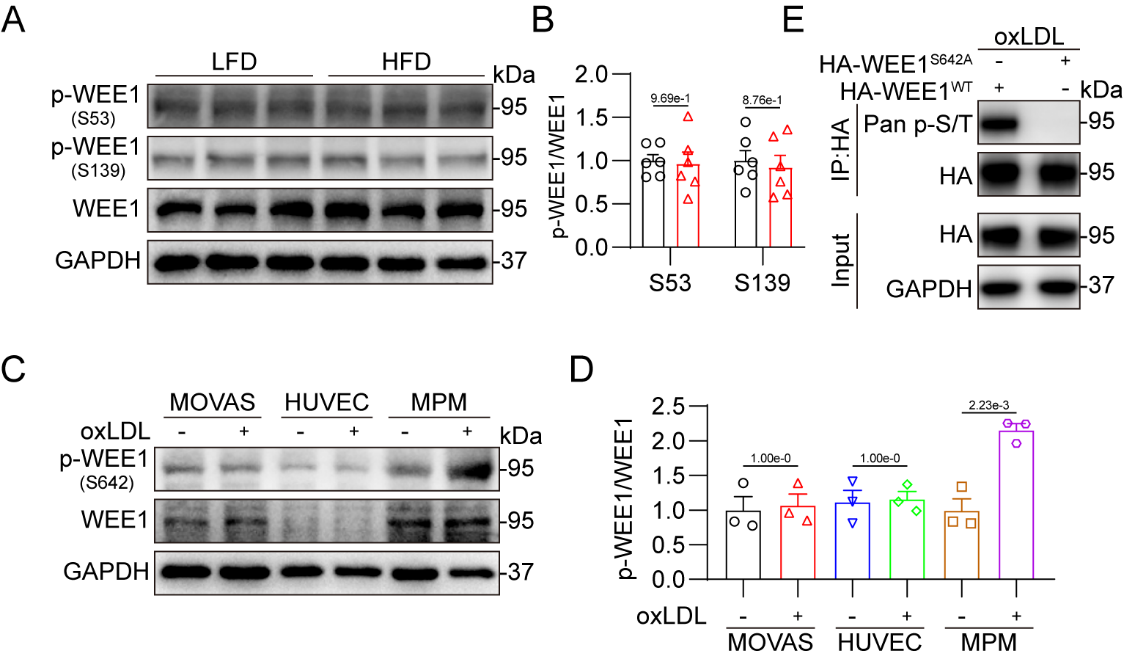
**

**Supplementary Figure S2. Macrophage WEE1 was phosphorylated at S642 in atherosclerotic plaques.** (A-B) Western blot analysis (A) and densitometric quantification (B) of p-WEE1 (S53), p-WEE1 (S139) in aortas of LFD and HFD-fed ApoE^-/-^ mice. GAPDH and WEE1 were used as loading controls (n=6). (C-D) Mouse aortic vascular smooth muscle cell (MOVAS), human umbilical vein endothelial cell (HUVEC) and mouse primary peritoneal macrophage (MPM) were challenged with or without oxLDL (50 μg/mL) for 120 min. Western blot analysis (C) and densitometric quantification (D) of p-WEE1. GAPDH and WEE1 were used as loading controls (n=3). (E) MPMs isolated from WEE1^MCKO^ mice were transfected with HA-WEE1^WT^ or HA-WEE1^S642A^ and challenged with oxLDL (50 μg/mL) for 120 min and then HA-WEE1^WT^ or HA-WEE1^S642A^ was immunoprecipitated by anti-HA antibody. Phosphorylated WEE1 was detected by immunoblotting using anti-Pan Phospho-Serine/Threonine antibody (Pan p-S/T). Data were shown as mean ± SEM. B, two-way ANOVA followed by Tukey's test; D, one-way ANOVA followed by Tukey's test.

**
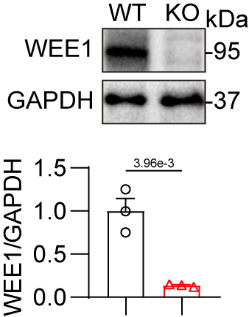
**

**Supplementary Figure S3. Knockout efficiency of WEE1 in MPMs.** Western blot analysis and densitometric quantification of WEE1 in MPMs isolated from WEE1^f/f^ and WEE1^MCKO^ mice. GAPDH was used as the loading control (n=3). Data were expressed as mean ± SEM. Student’s t-test.


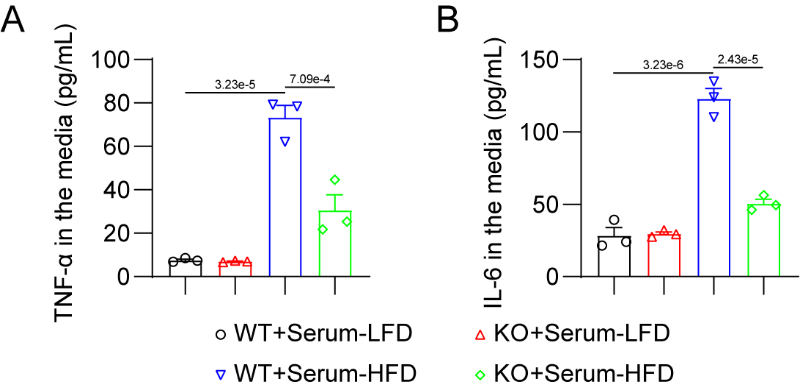


**Supplementary Figure S4.** **WEE1 deletion inhibits inflammatory response in macrophages challenged by mouse serum including hyperlipidemia.** (A-B) MPMs isolated from WEE1^f/f^ and WEE1^MCKO^ mice were challenged with serum (50 μL/mL) from ApoE^-/-^ mice fed a low (LFD) or high fat diet (HFD) for 24 h. Protein levels of TNF-α (A) and IL-6 (B) were analyzed using ELISA (n=3). Data were shown as mean ± SEM. A-B, one-way ANOVA followed by Tukey's test.

**
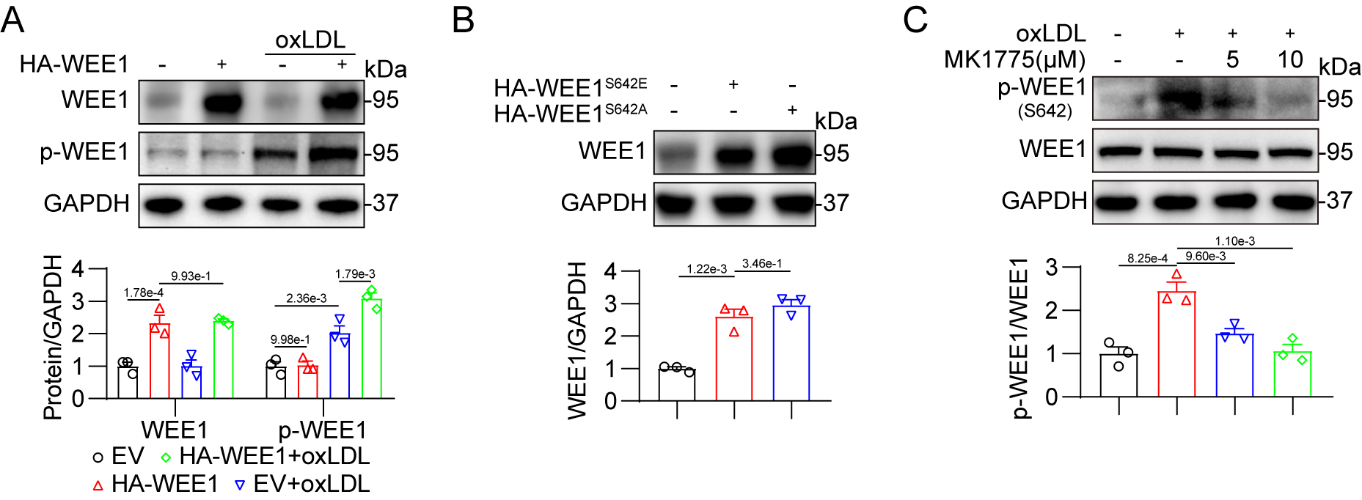
**

**Supplementary Figure S5. Effects of WEE1 activation in MPMs.** (A) MPMs transfected with HA-WEE1 or empty vector (EV) were challenged with oxLDL (50 μg/mL) for 2 h. Western blot analysis and densitometric quantification of WEE1 and p-WEE1. GAPDH was used as the loading control (n=3). (B) Western blot analysis and densitometric quantification of WEE1 in MPMs transfected with EV, HA-WEE1^S642E^ or HA-WEE1^S642A^ for 24 h. GAPDH was used as the loading control (n=3). (C) MPMs were pretreated with MK1775 (5 and 10 μM) or vehicle (DMSO, 1‰) for 1 h, followed by exposure of oxLDL (50 μg/mL) for 2 h. Western blot analysis of p-WEE1. GAPDH and WEE1 were used as loading controls (n=3). Data were expressed as mean ± SEM. A, two-way ANOVA followed by Tukey's test; B-C, one-way ANOVA followed by Tukey's test.


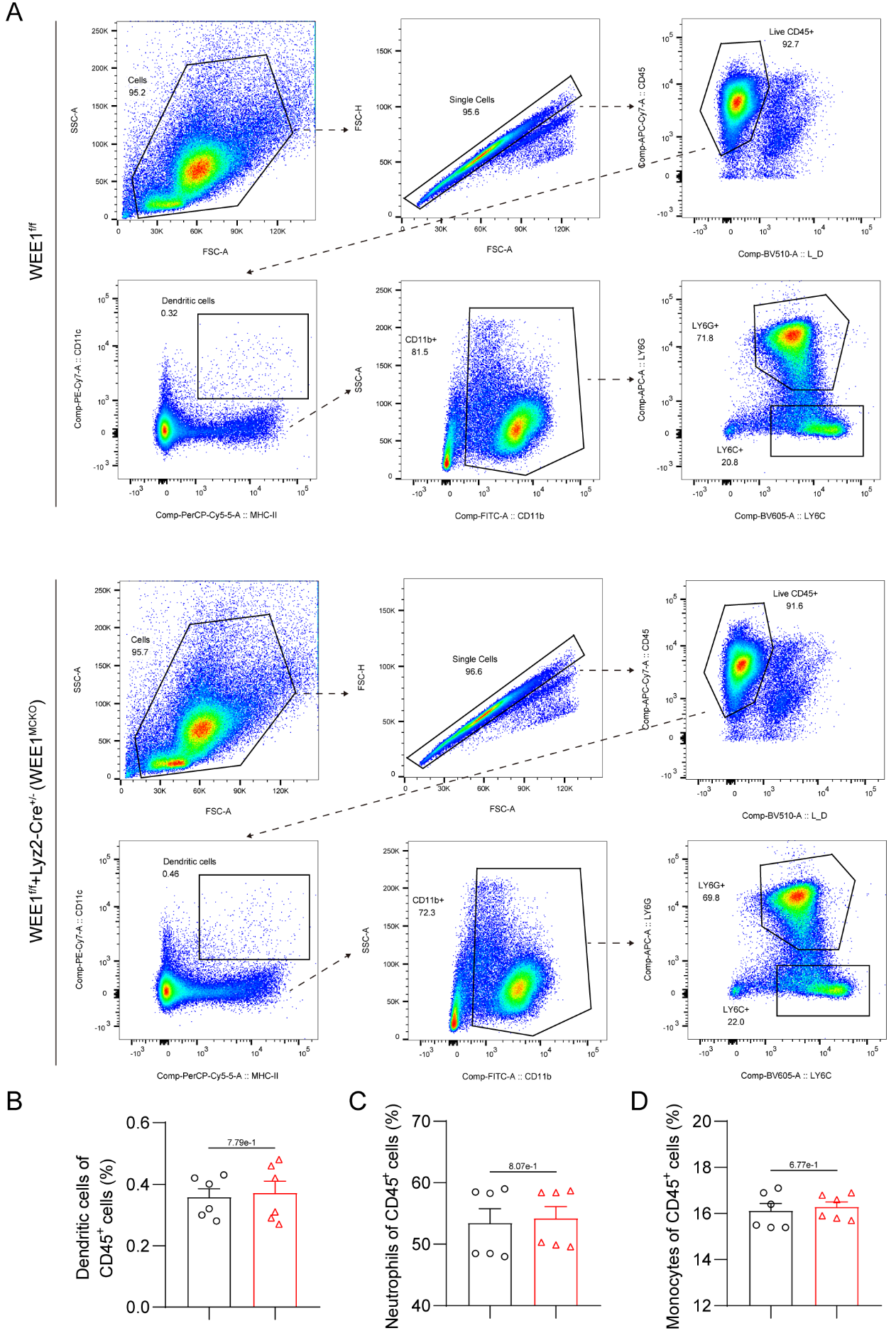


**Supplementary Figure S6.** **Macrophage-specific WEE1 deletion does not affect the composition of myeloid progenitor subsets in bone marrow.** (A-D) Representative flow cytometric images (A) and quantification of dendritic cells (B, CD45^+^MHC-II^+^CD11c^+^), neutrophils (C, CD45^+^CD11b^+^Ly6G^+^) and monocytes (D, CD45^+^CD11b^+^Ly6C^+^) in the bone marrow cells from WEE1^f/f^ or WEE1^MCKO^ mice. Data were shown as mean ± SEM. B-D, Student’s t-test.


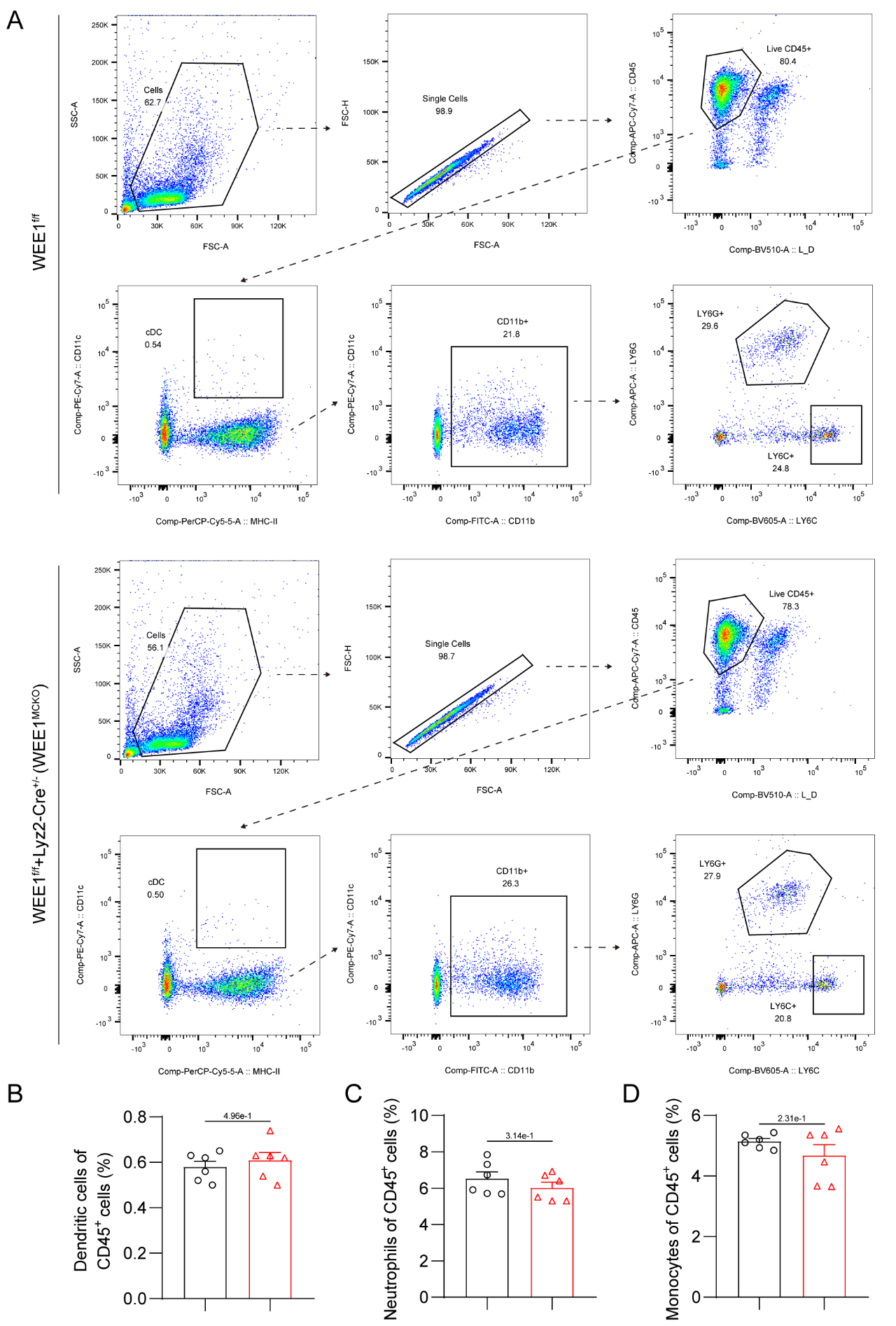


**Supplementary Figure S7. Macrophage-specific WEE1 deletion does not affect the composition of myeloid cells subsets in circulation.** (A-D) Representative flow cytometric images (A) and quantification of dendritic cells (B, CD45^+^MHC-II^+^CD11c^+^), neutrophils (C, CD45^+^CD11b^+^Ly6G^+^) and monocytes (D, CD45^+^CD11b^+^Ly6C^+^) in the peripheral blood cells from WEE1^f/f^ or WEE1^MCKO^ mice. Data were shown as mean ± SEM. B-D, Student’s t-test.


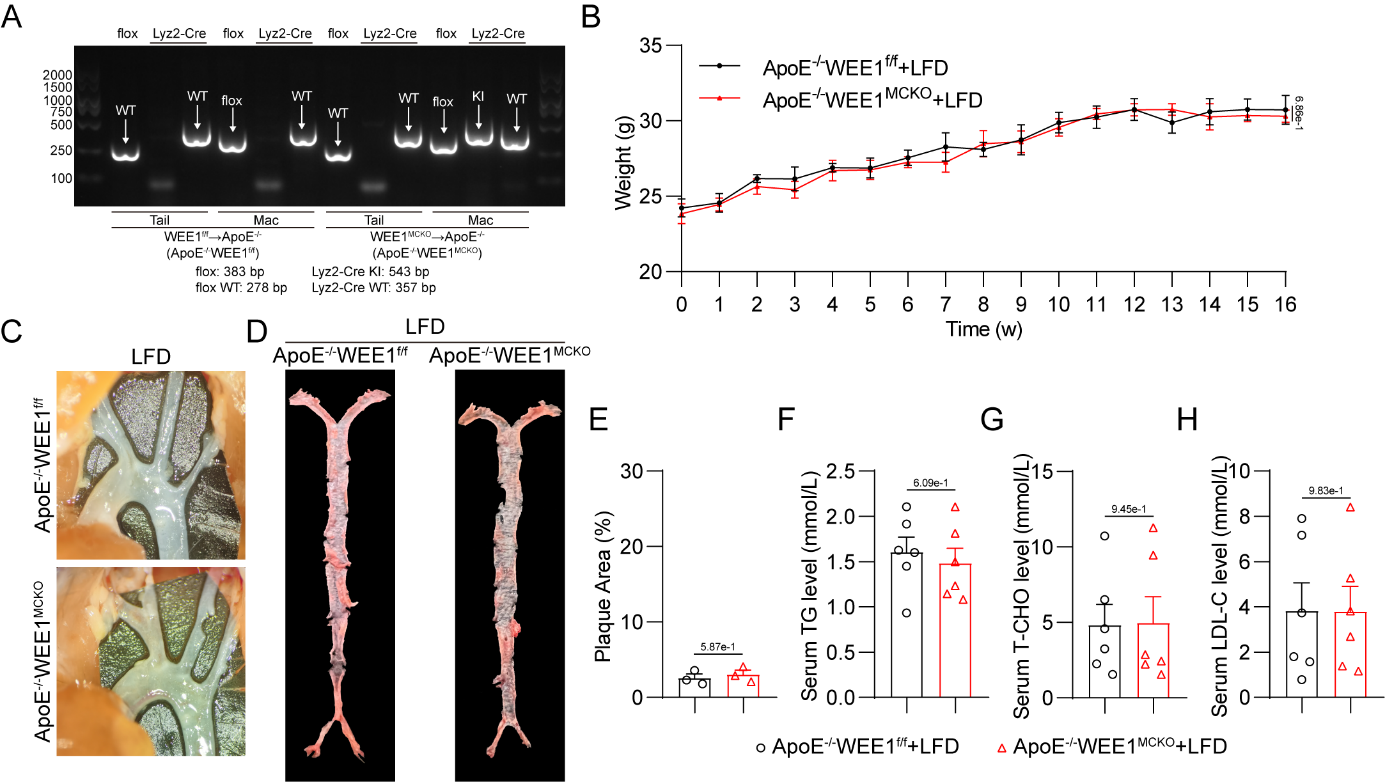


**Supplementary Figure S8. Macrophage-specific WEE1 deletion does not affect the viability, normal vascular development, and serum lipid profile in LFD-fed ApoE^-/-^ mice.** (A) To identify the genotypes of ApoE^-/-^WEE1^f/f^ and ApoE^-/-^WEE1^MCKO^ mice after bone marrow transplantation, cDNA was extracted from mouse tail or MPMs and the primers for identification of ApoE^-/-^ WEE1^f/f^ and ApoE^-/-^ WEE1^MCKO^ mice were used for PCR assay. Mac, macrophage. (B) ApoE^-/-^ mice were irradiated and administered bone marrow cells (BMCs) from either WEE1^f/f^ or WEE1^MCKO^ mice. After 3 weeks, these mice were fed a low-fat diet (LFD) for 16 weeks. Average body weights of mice recoded each week for the duration of the animal experiment (n=6). (C) Representative images of plaque lesion in aortic arches. (D-E) Representative en face Oil Red O staining (D) and quantification (E) of Oil Red O-positive plaque lesion area in aortas. Plaque area was defined as percentage of total surface area of the aorta (n=3). (F-H) Serum levels of triglycerides (TG) (F), total cholesterol (T-CHO) (G), low-density lipoproteins (LDL-C) (H) were examined using commercial kits (n=6). Data were expressed as mean ± SEM. B and E-H, Student’s t-test.


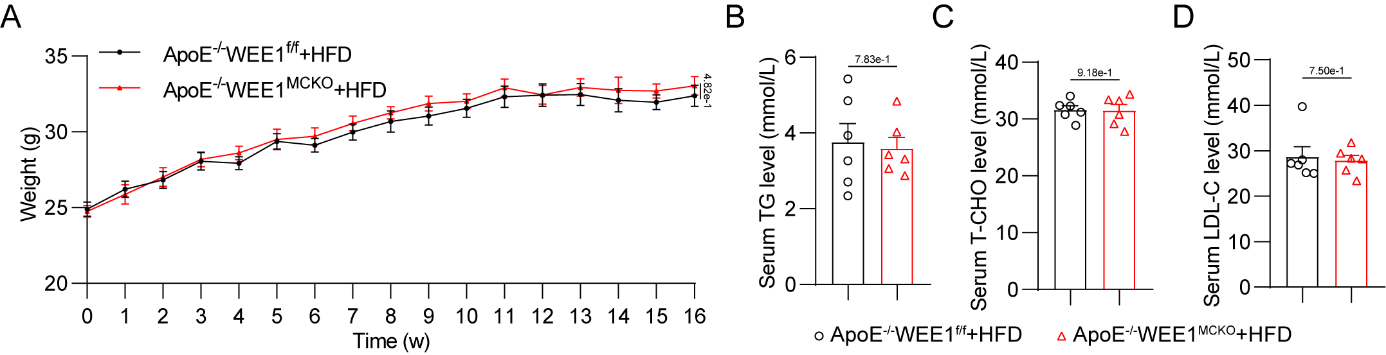


**Supplementary Figure S9. Macrophage-specific WEE1 deletion does not affect serum lipid profile in HFD-fed ApoE^-/-^ mice.** (A) Average body weights of mice recoded each week for the duration of the animal experiment (n=12). (B-D) Serum levels of triglycerides (TG) (B), total cholesterol (T-CHO) (C), low-density lipoproteins (LDL-C) (D) were examined using commercial kits (n=6). Data were expressed as mean ± SEM. A-D, Student’s t-test.


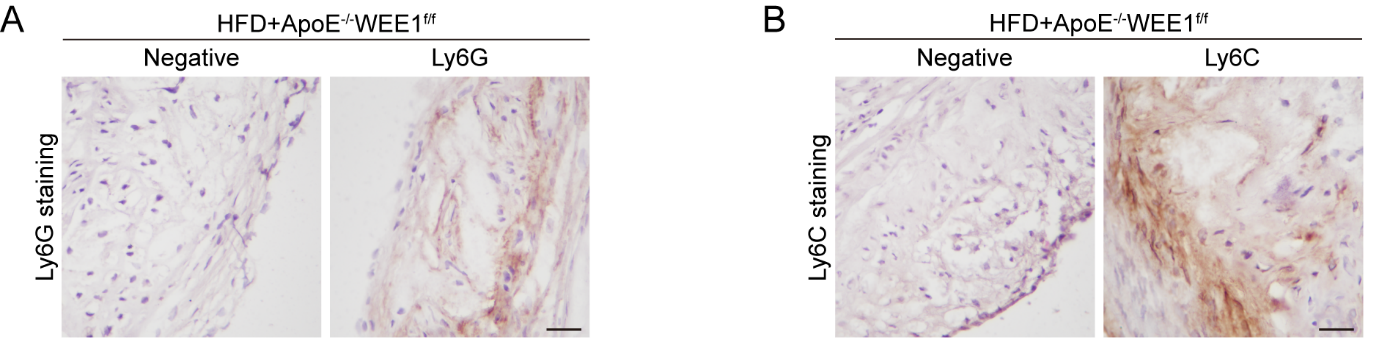


**Supplementary Figure S10.** Representative immunohistochemistry staining images of Ly6G (A) and Ly6C (B) in aortic roots (scale bar=25 μm).


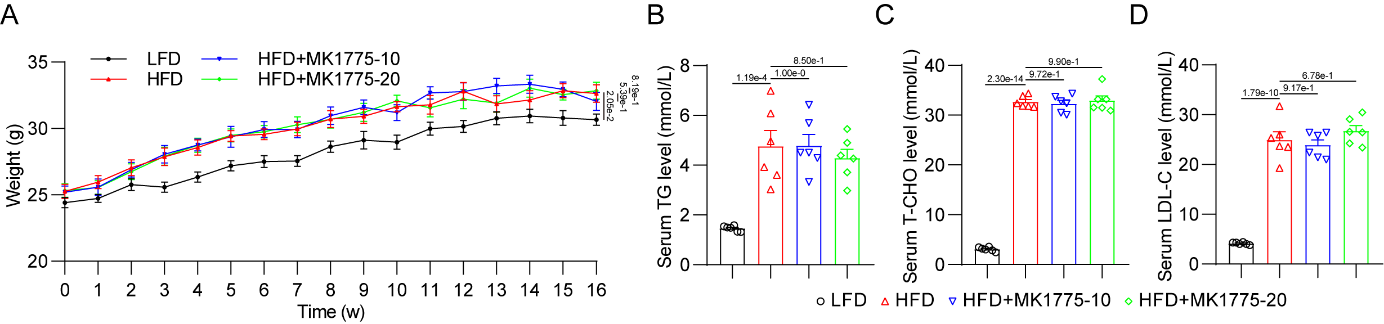
**Supplementary Figure S11. MK1775 does not affect serum lipid profile in HFD-fed ApoE^-/-^ mice.** (A) Average body weights of mice recoded each week for the duration of the animal experiment (n=12). (B-D) Serum levels of triglycerides (TG) (B), total cholesterol (T-CHO) (C), low-density lipoproteins (LDL-C) (D) were examined using commercial kits (n=6). Data were expressed as mean ± SEM. A-D, one-way ANOVA followed by Tukey's test.

**
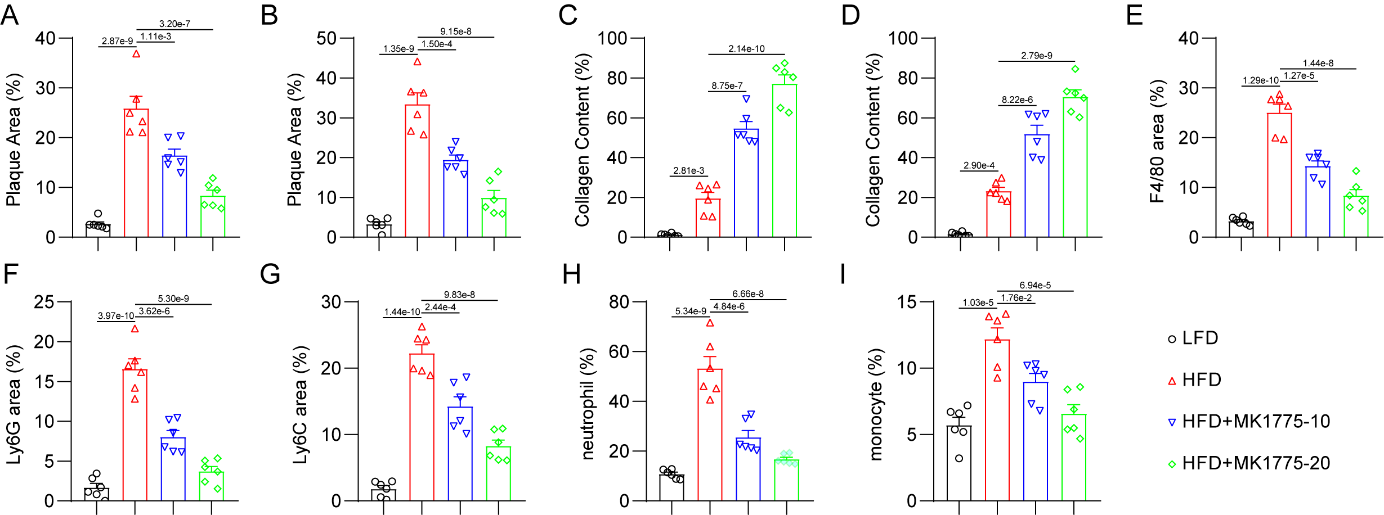
**

**Supplementary Figure S12.** **Quantification for Figure 5B-I.** (A-I) Quantification for Figure 5B-I, respectively (n=6). Plaques were analyzed from first appearance of intact three sinus valves. Data were expressed as mean ± SEM. A-I, one-way ANOVA followed by Tukey's test.


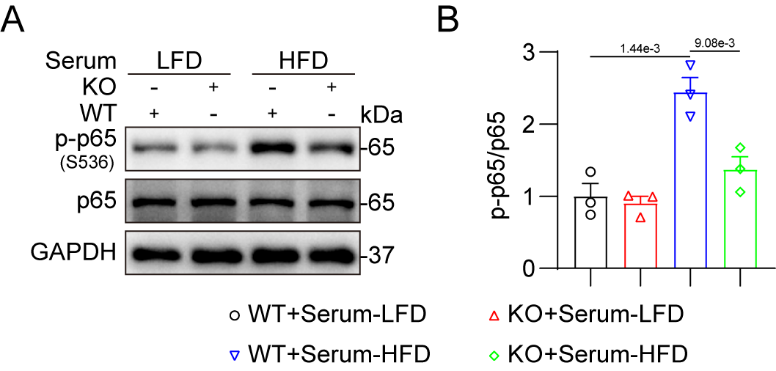


**Supplementary Figure S13. WEE1 deletion inhibits p65 activation in macrophages challenged by mouse serum including hyperlipidemia.** (A-B) MPMs isolated from WEE1^f/f^ and WEE1^MCKO^ mice were challenged with serum (50 μL/mL) from ApoE^-/-^ mice fed a low (LFD) or high fat diet (HFD) for 1 h. Western blot analysis (A) and densitometric quantification (B) of p-p65. GAPDH and p65 were used as loading controls (n=3). Data were shown as mean ± SEM. B, one-way ANOVA followed by Tukey's test.

**
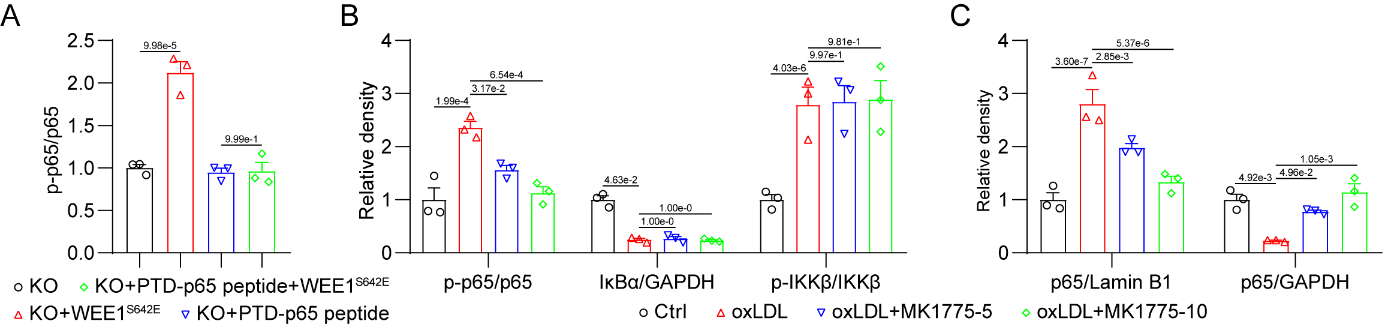
**

**Supplementary Figure S14. Quantification for Figure 6Q-S.** (A-C) Quantification for Figure 6Q-S, respectively (n=3). Data were expressed as mean ± SEM. A, one-way ANOVA followed by Tukey's test; B-C, two-way ANOVA followed by Tukey's test.

**
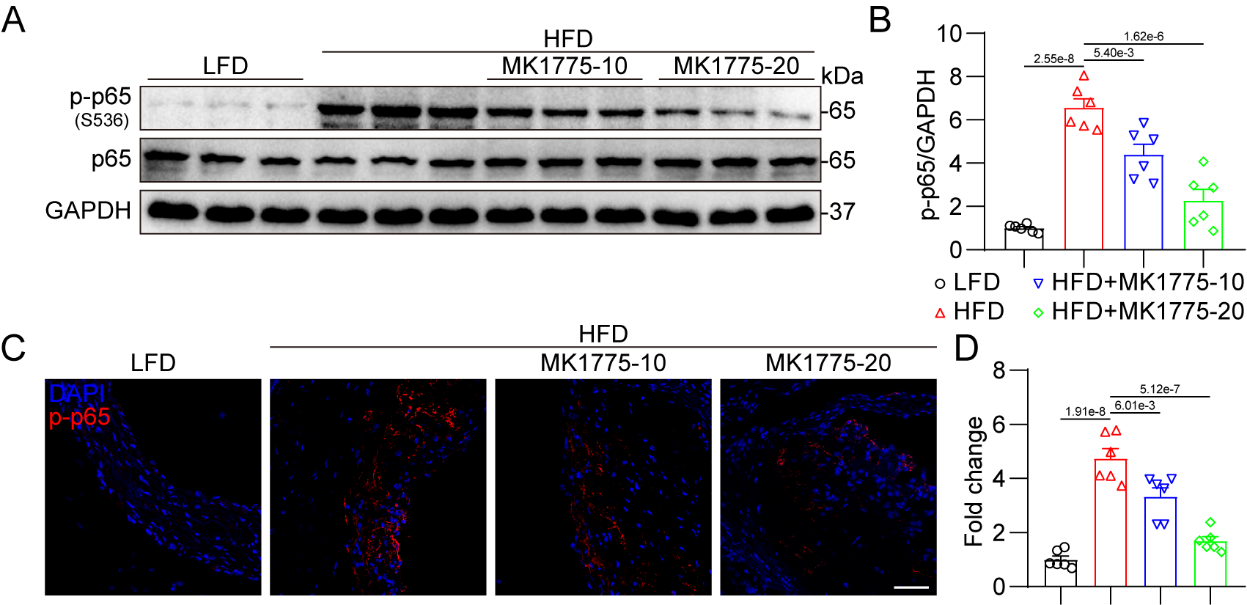
**

**Supplementary Figure S15. Pharmacological inhibitor of WEE1 kinase activity inhibits** **p65 phosphorylation in atherosclerotic lesions.** (A-B) Western blot analysis (A) and densitometric quantification (B) of p-p65 in aortic tissues of the mice. GAPDH and p65 were used as loading controls (n=6). (C-D) Representative immunofluorescence staining images (C) and quantification (D) of p-p65 (red) in aortic roots. Tissues were counterstained with DAPI (blue). Scale bar=50 μm, n=6. Plaques were analyzed from first appearance of intact three sinus valves. Data were expressed as mean ± SEM. B and D, one-way ANOVA followed by Tukey's test.

**
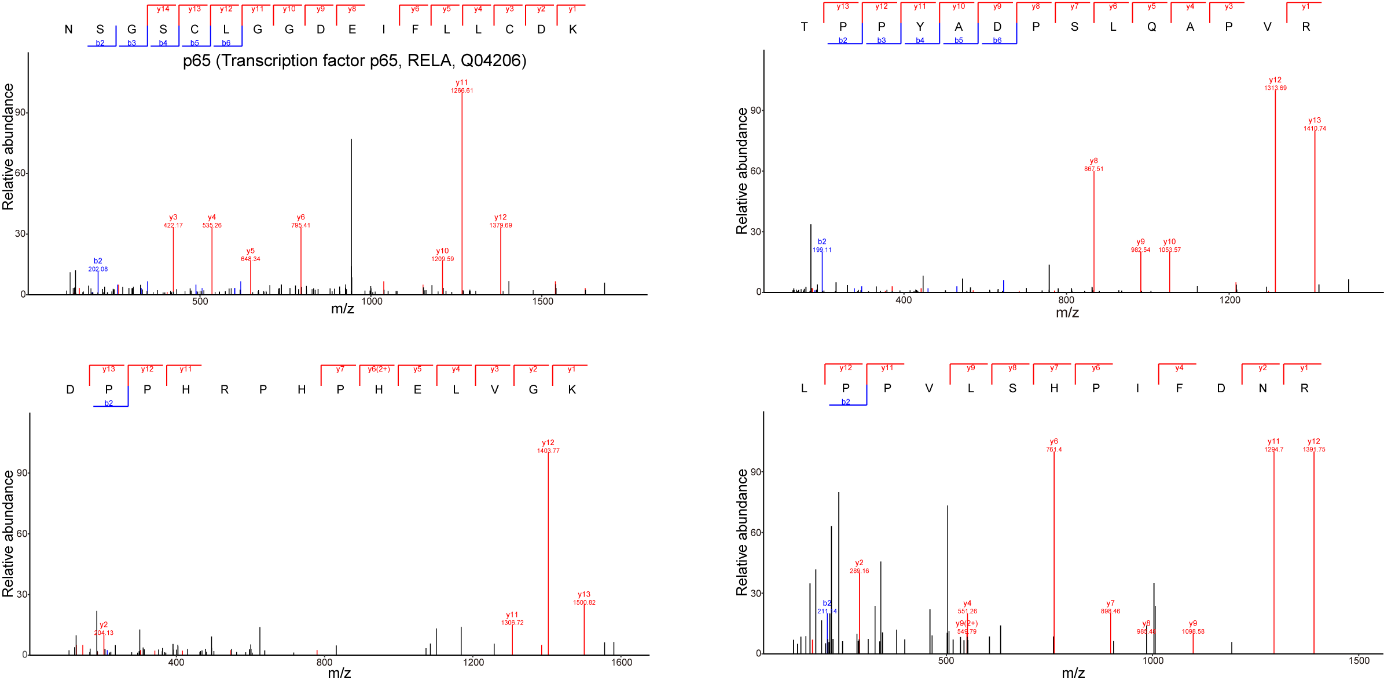
**

**Supplementary Figure S16. Second-order spectrums of the peptide of p65.**

**
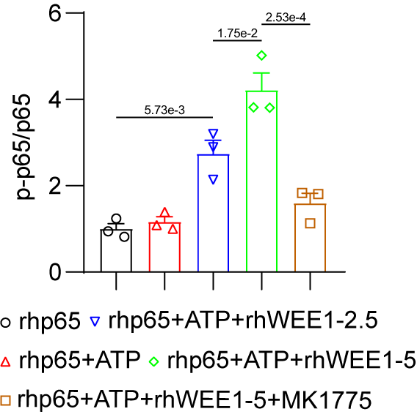
**

**Supplementary Figure S17. Quantification for Figure 7I.** Data were expressed as mean ± SEM. n=3, one-way ANOVA followed by Tukey's test.

**Supplementary Table S1.** The DEGs of GO term-inflammatory response

| Gene  name | log2(FC) | pValue | FPKM.KO  +oxLDL1 | FPKM.KO  +oxLDL2 | FPKM.KO  +oxLDL3 | FPKM.WT  +oxLDL1 | FPKM.WT  +oxLDL2 | FPKM.WT  +oxLDL3 |
| --- | --- | --- | --- | --- | --- | --- | --- | --- |
| **Il1f6** | -13.97 | 3.43E-14 | 0 | 0 | 0 | 1.45 | 1.50 | 1.88 |
| **Cxcl11** | -12.03 | 1.12E-14 | 0 | 0 | 0 | 0.31 | 0.45 | 0.50 |
| **Fpr3** | -11.80 | 2.54E-08 | 0 | 0 | 0 | 0.34 | 0.42 | 0.30 |
| **Il17a** | -10.00 | 2.72E-03 | 0 | 0 | 0 | 0.09 | 0.18 | 0.04 |
| **Il23a** | -9.72 | 1.30E-04 | 0 | 0 | 0 | 0.09 | 0.02 | 0.14 |
| **Nos2** | -9.21 | 4.37E-48 | 0.66 | 0.83 | 2.58 | 808.75 | 805.74 | 793.01 |
| **Cxcl10** | -9.03 | 4.91E-107 | 0.68 | 0.66 | 1.52 | 475.78 | 487.12 | 530.83 |
| **Cxcl9** | -8.89 | 6.06E-61 | 0.04 | 0.02 | 0.09 | 21.44 | 24.75 | 23.97 |
| **Il6** | -8.82 | 1.17E-11 | 0 | 0.10 | 0.17 | 37.83 | 39.39 | 43.85 |
| **Fpr1** | -8.59 | 3.88E-89 | 0.99 | 1.26 | 2.60 | 600.39 | 637.93 | 634.67 |
| **Gbp5** | -8.58 | 4.45E-93 | 0.43 | 0.75 | 1.27 | 301.48 | 315.13 | 321.52 |
| **Il1b** | -8.54 | 1.00E-65 | 0.50 | 0.88 | 1.78 | 384.70 | 386.24 | 401.65 |
| **Ptgs2** | -8.36 | 4.50E-171 | 0.51 | 0.79 | 1.12 | 268.47 | 261.02 | 265.39 |
| **Cxcl3** | -8.25 | 2.00E-30 | 0.01 | 0.11 | 0.15 | 31.51 | 24.86 | 26.01 |
| **Fpr2** | -8.15 | 0.00E+00 | 1.22 | 1.81 | 2.13 | 478.15 | 472.10 | 512.29 |
| **Ccl12** | -8.06 | 2.22E-13 | 0 | 0.16 | 0.40 | 45.03 | 53.11 | 53.00 |
| **Ccl7** | -7.86 | 1.72E-266 | 2.75 | 2.75 | 4.22 | 662.86 | 787.26 | 811.45 |
| **Ccl5** | -7.82 | 1.37E-124 | 2.76 | 2.17 | 4.68 | 714.47 | 715.55 | 733.35 |
| **Cxcl2** | -7.70 | 0.00E+00 | 0.72 | 0.53 | 0.71 | 131.33 | 132.45 | 145.11 |
| **Cxcl1** | -7.24 | 1.85E-50 | 0.05 | 0.07 | 0.18 | 17.96 | 10.86 | 17.46 |
| **Tnfa** | -7.17 | 0.00E+00 | 4.46 | 5.57 | 6.60 | 767.66 | 805.54 | 819.50 |
| **Ccl2** | -7.14 | 0.00E+00 | 5.31 | 5.56 | 5.84 | 719.04 | 797.86 | 848.69 |
| **Lta** | -7.00 | 3.69E-06 | 0 | 0 | 0.07 | 3.78 | 3.03 | 2.35 |
| **Trex1** | -6.85 | 1.24E-16 | 0.93 | 0.42 | 0.09 | 30.80 | 91.49 | 43.10 |
| **Cd40** | -6.66 | 0.00E+00 | 0.95 | 1.48 | 1.29 | 118.05 | 123.03 | 136.11 |
| **Il27** | -6.42 | 1.36E-46 | 0.39 | 0.38 | 1.02 | 43.63 | 51.64 | 57.77 |
| **Cxcl5** | -6.41 | 4.25E-05 | 0.05 | 0 | 0 | 1.47 | 0.89 | 1.72 |
| **Chil1** | -6.40 | 9.66E-14 | 0.02 | 0 | 0 | 0.26 | 0.64 | 0.59 |
| **Mefv** | -5.96 | 0.00E+00 | 1.57 | 1.67 | 1.89 | 102.49 | 108.14 | 108.00 |
| **Ccl3** | -5.07 | 0.00E+00 | 34.64 | 35.16 | 34.51 | 1160.73 | 1132.70 | 1206.72 |
| Nfkbiz | -4.87 | 0.00E+00 | 1.61 | 1.38 | 1.62 | 43.17 | 45.97 | 45.83 |
| Tnfsf4 | -4.83 | 7.77E-07 | 0 | 0.04 | 0 | 0.21 | 0.34 | 0.46 |
| Il1a | -4.77 | 0.00E+00 | 25.82 | 26.64 | 28.08 | 734.80 | 740.67 | 728.74 |
| Smpdl3b | -4.68 | 3.67E-244 | 0.85 | 0.66 | 0.51 | 17.85 | 16.25 | 17.77 |
| Nlrp3 | -4.56 | 0.00E+00 | 4.50 | 4.07 | 4.33 | 101.53 | 99.51 | 103.26 |
| Ccl4 | -4.50 | 0.00E+00 | 6.58 | 5.49 | 5.89 | 118.83 | 144.03 | 142.52 |
| Il1rn | -4.25 | 0.00E+00 | 12.30 | 12.37 | 12.93 | 210.98 | 259.34 | 246.59 |
| Casp4 | -4.11 | 0.00E+00 | 3.17 | 3.48 | 3.14 | 50.99 | 57.13 | 60.32 |
| Il2ra | -3.93 | 2.45E-30 | 0.03 | 0.03 | 0.04 | 0.52 | 0.70 | 0.46 |
| Il18 | -3.90 | 0.00E+00 | 3.24 | 3.50 | 3.95 | 48.70 | 57.05 | 53.73 |
| Tlr2 | -3.85 | 0.00E+00 | 17.88 | 17.11 | 17.27 | 251.42 | 254.08 | 247.98 |
| Cd14 | -3.83 | 0.00E+00 | 24.69 | 22.66 | 23.52 | 343.18 | 338.41 | 328.36 |
| Ccl9 | -3.83 | 0.00E+00 | 14.22 | 14.03 | 12.23 | 184.58 | 187.96 | 204.37 |
| Nod2 | -3.82 | 0.00E+00 | 1.03 | 1.09 | 1.14 | 15.51 | 16.06 | 14.69 |
| Ccl22 | -3.78 | 2.57E-81 | 0.30 | 0.27 | 0.25 | 3.92 | 3.46 | 3.76 |
| Aif1 | -3.77 | 6.08E-120 | 1.02 | 1.29 | 0.81 | 11.95 | 15.49 | 15.18 |
| Il1f9 | -3.46 | 8.78E-59 | 0.85 | 0.40 | 0.90 | 8.36 | 7.78 | 7.40 |
| Tlr3 | -3.43 | 1.48E-283 | 1.33 | 1.35 | 1.61 | 13.96 | 16.87 | 15.35 |
| Tnfaip3 | -3.30 | 0.00E+00 | 11.28 | 11.06 | 12.29 | 109.95 | 118.03 | 112.45 |
| Hck | -3.03 | 0.00E+00 | 29.82 | 31.85 | 31.53 | 258.18 | 252.27 | 248.72 |
| Lacc1 | -2.96 | 0.00E+00 | 19.82 | 20.42 | 21.29 | 155.25 | 156.89 | 167.08 |
| Cd163 | -2.96 | 4.94E-19 | 0.08 | 0.04 | 0.08 | 0.39 | 0.59 | 0.62 |
| Axl | -2.85 | 0.00E+00 | 8.60 | 9.47 | 9.71 | 68.01 | 64.60 | 67.47 |
| Zc3h12a | -2.69 | 0.00E+00 | 9.36 | 9.44 | 9.87 | 64.53 | 58.56 | 62.52 |
| Tnip1 | -2.55 | 0.00E+00 | 34.78 | 33.31 | 34.54 | 204.58 | 203.68 | 192.53 |
| Mapkapk2 | -2.49 | 0.00E+00 | 56.89 | 55.75 | 57.91 | 325.24 | 306.47 | 324.27 |
| Tlr1 | -2.43 | 0.00E+00 | 15.50 | 16.70 | 15.81 | 85.80 | 85.04 | 87.93 |
| Sema7a | -2.36 | 1.81E-03 | 0.07 | 0 | 0.06 | 0.11 | 0.28 | 0.27 |
| C5ar1 | -2.34 | 9.33E-280 | 10.87 | 11.26 | 12.36 | 56.07 | 61.18 | 56.98 |
| Il15 | -2.23 | 1.47E-160 | 4.77 | 5.59 | 4.91 | 23.04 | 23.09 | 25.60 |
| Myd88 | -2.21 | 0.00E+00 | 16.12 | 17.01 | 17.07 | 78.75 | 76.99 | 76.52 |
| Relb | -2.18 | 2.14E-170 | 2.46 | 2.48 | 2.40 | 11.22 | 10.62 | 11.45 |
| Il18rap | -2.12 | 3.70E-07 | 0.04 | 0.05 | 0.04 | 0.19 | 0.12 | 0.26 |
| Cxcr6 | -2.09 | 1.40E-05 | 0.22 | 0.06 | 0.11 | 0.70 | 0.57 | 0.44 |
| Cybb | -2.09 | 0.00E+00 | 202.85 | 217.33 | 204.17 | 863.77 | 885.30 | 902.51 |
| Thbs1 | -2.06 | 4.99E-199 | 22.90 | 26.52 | 29.07 | 104.21 | 114.57 | 108.26 |
| Csf1 | -2.06 | 5.00E-179 | 3.92 | 3.48 | 3.56 | 15.84 | 14.24 | 15.50 |
| Cmklr1 | -2.03 | 1.23E-215 | 13.65 | 13.87 | 15.77 | 62.36 | 58.34 | 55.71 |
| C4b | -2.01 | 1.00E-173 | 2.73 | 2.63 | 2.87 | 11.60 | 10.89 | 10.61 |
| Ccrl2 | -2.01 | 0.00E+00 | 48.50 | 49.17 | 44.22 | 184.31 | 190.28 | 195.03 |
| Lyn | -1.97 | 0.00E+00 | 27.47 | 30.27 | 30.83 | 113.07 | 116.75 | 117.19 |
| Tnfrsf1b | -1.94 | 0.00E+00 | 239.02 | 247.53 | 261.57 | 989.50 | 941.84 | 930.91 |
| Kdm6b | -1.92 | 5.48E-36 | 1.12 | 0.91 | 1.51 | 5.60 | 3.98 | 3.80 |
| Themis2 | -1.88 | 3.09E-270 | 30.95 | 32.42 | 34.31 | 115.50 | 118.49 | 125.30 |
| Ccr5 | -1.87 | 2.40E-103 | 2.65 | 2.87 | 2.49 | 8.31 | 10.40 | 10.51 |
| Cers6 | -1.86 | 0.00E+00 | 24.78 | 25.44 | 25.51 | 92.48 | 93.39 | 88.86 |
| Tlr7 | -1.82 | 0.00E+00 | 33.96 | 36.90 | 35.50 | 121.64 | 128.48 | 125.79 |
| Ccr7 | -1.81 | 9.19E-09 | 0.31 | 0.24 | 0.41 | 0.84 | 1.20 | 1.35 |
| Chst1 | -1.79 | 6.73E-04 | 0.04 | 0.07 | 0.17 | 0.34 | 0.33 | 0.29 |
| Tlr9 | -1.76 | 1.12E-110 | 4.71 | 4.20 | 4.90 | 16.15 | 14.67 | 15.91 |
| Tlr11 | -1.76 | 7.22E-04 | 0.07 | 0.03 | 0.09 | 0.21 | 0.22 | 0.20 |
| Gsdmd | -1.73 | 1.85E-189 | 16.20 | 15.81 | 14.70 | 51.38 | 49.82 | 53.95 |
| Ciita | -1.70 | 5.37E-72 | 0.83 | 0.89 | 0.86 | 3.11 | 2.72 | 2.59 |
| Pla2g7 | -1.69 | 7.73E-243 | 88.75 | 91.63 | 85.17 | 267.83 | 290.22 | 301.35 |
| S1pr3 | -1.59 | 1.05E-05 | 0.16 | 0.10 | 0.10 | 0.23 | 0.43 | 0.41 |
| Pik3cg | -1.55 | 6.02E-218 | 11.67 | 11.58 | 11.27 | 32.13 | 35.10 | 33.66 |
| Pxk | -1.51 | 1.43E-101 | 6.55 | 6.10 | 7.07 | 18.99 | 19.51 | 17.50 |
| Pla2g2d | -1.50 | 2.51E-03 | 0.07 | 0.09 | 0.14 | 0.27 | 0.25 | 0.33 |
| Stat3 | -1.48 | 2.00E-261 | 13.92 | 13.59 | 14.08 | 39.11 | 38.55 | 38.03 |
| Rela | -1.42 | 3.65E-134 | 20.41 | 23.00 | 22.22 | 63.19 | 55.47 | 57.14 |
| Nlrc4 | -1.40 | 2.57E-64 | 2.64 | 2.79 | 2.80 | 7.40 | 7.80 | 6.55 |
| S100a8 | -1.29 | 2.15E-07 | 1.68 | 1.40 | 1.33 | 2.92 | 3.78 | 4.10 |
| Rps6ka4 | -1.23 | 4.55E-73 | 7.53 | 8.32 | 8.88 | 20.07 | 18.06 | 19.78 |
| Pf4 | -1.22 | 3.60E-52 | 20.54 | 19.50 | 17.52 | 43.94 | 46.99 | 43.61 |
| Olr1 | -1.20 | 3.60E-24 | 2.46 | 2.35 | 1.81 | 4.64 | 4.82 | 5.73 |
| Cxcl13 | -1.15 | 6.55E-36 | 8.12 | 8.49 | 8.36 | 18.10 | 20.27 | 16.89 |
| Ly86 | -1.01 | 5.10E-67 | 9.90 | 11.13 | 10.49 | 20.04 | 21.38 | 22.06 |

**Supplementary Table S2.** Mass spectrometry peptide sequences and scores of p65

| Sequence | Protein | Score |
| --- | --- | --- |
| NSGSCLGGDEIFLLCDK | p65 | 130.2 |
| TPPYADPSLQAPVR | p65 | 113.22 |
| DPPHRPHPHELVGK | p65 | 46.797 |
| LPPVLSHPIFDNR | p65 | 37.013 |

**Supplementary Table S3.** Clinical parameters for the human samples

| No. | Nationality | Age (years) | Sex | Plaque size (mm*mm) |
| --- | --- | --- | --- | --- |
| 1 | Chinese | 74 | male | 12.2*2.5 |
| 2 | Chinese | 69 | male | 9.9*1.6 |
| 3 | Chinese | 74 | male | 8.2*1.9 |

**Supplementary Table S4.** Primer sequences for PCR genotyping analysis

| **Gene** | **Species** | **Sequence** |
| --- | --- | --- |
| *Flox* | Mouse | GAACTCATGGATATTCCAGGTTGC GGGCTTATTTCCCGTGTTAAAC |
| *Lyz2-Cre KI* | Mouse | AGTGCTGAAGTCCATAGATCGG CTGATTCTCCTCATCACCAGG |
| *Lyz2-Cre WT* | Mouse | AGTGCTGAAGTCCATAGATCGG GTCACTCACTGCTCCCCTGT |

**Supplementary Table S5.** Primer sequences for real-time qPCR assay

| **Gene** | **Species** | **Sequence** |
| --- | --- | --- |
| *Tnf-α* | Mouse | TGATCCGCGACGTGGAA ACCGCCTGGAGTTCTGGAA |
| *Il-6* | Mouse | GAGGATACCACTCCCAACAGACC AAGTGCATCATCGTTGTTCATACA |
| *Il-1β* | Mouse | GCAACTGTTCCTGAACTCAACT ATCTTTTGGGGTCCGTCAACT |
| *Il-1f6* | Mouse | GCAGCATCACCTTCGCTTAGA CAGATATTGGCATGGGAGCAAG |
| *Il-17α* | Mouse | TTTAACTCCCTTGGCGCAAAA CTTTCCCTCCGCATTGACAC |
| *Il-23α* | Mouse | ATGCTGGATTGCAGAGCAGTA ACGGGGCACATTATTTTTAGTCT |
| *Il-27* | Mouse | CTGTTGCTGCTACCCTTGCTT CACTCCTGGCAATCGAGATTC |
| *Ccl2* | Mouse | TTAAAAACCTGGATCGGAACCAA GCATTAGCTTCAGATTTACGGGT |
| *Ccl3* | Mouse | TTCTCTGTACCATGACACTCTGC CGTGGAATCTTCCGGCTGTAG |
| *Ccl5* | Mouse | GCTGCTTTGCCTACCTCTCC TCGAGTGACAAACACGACTGC |
| *Ccl7* | Mouse | GCTGCTTTCAGCATCCAAGTG CCAGGGACACCGACTACTG |
| *Ccl12* | Mouse | ATTTCCACACTTCTATGCCTCCT ATCCAGTATGGTCCTGAAGATCA |
| *Cxcl1* | Mouse | CTGGGATTCACCTCAAGAACATC CAGGGTCAAGGCAAGCCTC |
| *Cxcl2* | Mouse | CCAACCACCAGGCTACAGG GCGTCACACTCAAGCTCTG |
| *Cxcl3* | Mouse | TGAGACCATCCAGAGCTTGACG CCTTGGGGGTTGAGGCAAACTT |
| *Cxcl5* | Mouse | TCCAGCTCGCCATTCATGC TTGCGGCTATGACTGAGGAAG |
| *Cxcl9* | Mouse | TCCTTTTGGGCATCATCTTCC TTTGTAGTGGATCGTGCCTCG |
| *Cxcl10* | Mouse | CCAAGTGCTGCCGTCATTTTC GGCTCGCAGGGATGATTTCAA |
| *Cxcl11* | Mouse | GGCTTCCTTATGTTCAAACAGGG GCCGTTACTCGGGTAAATTACA |
| *Icam1* | Mouse | GTGATGCTCAGGTATCCATCCA CACAGTTCTCAAAGCACAGCG |
| *Vcam1* | Mouse | AGTTGGGGATTCGGTTGTTCT CCCCTCATTCCTTACCACCC |
| *Actb* | Mouse | GGCTGTATTCCCCTCCATCG CCAGTTGGTAACAATGCCATGT |
| *Rn18s* | Mouse | AGTCCCTGCCCTTTGTACACA CGATCCGAGGGCCTCACT |
